# Nanotherapy of Glioblastoma—Where Hope Grows

**DOI:** 10.3390/ijms26051814

**Published:** 2025-02-20

**Authors:** Jan Grzegorzewski, Maciej Michalak, Maria Wołoszczuk, Magdalena Bulicz, Aleksandra Majchrzak-Celińska

**Affiliations:** 1 The Student Scientific Society of Poznan University of Medical Sciences, 60-806 Poznań, Poland; j.grzegorzewski2001@gmail.com (J.G.); michalakm500@gmail.com (M.M.); woloszczukmaria2@gmail.com (M.W.); m.j.bulicz@gmail.com (M.B.); 2 Department of Pharmaceutical Biochemistry, Poznan University of Medical Sciences, Rokietnicka 3, 60-806 Poznań, Poland

**Keywords:** nanoparticles, nanotherapy, glioblastoma, liposomes, lipid nanocapsules, nanodiscs, dendrimers, metallic nanoparticles, silica nanoparticles, exosome

## Abstract

Localization in the central nervous system, diffuse growth, the presence of stem cells, and numerous resistance mechanisms, all make glioblastoma (GBM) an incurable tumor. The standard treatment of GBM consisting of surgery; radio- and chemotherapy with temozolomide provides insufficient therapeutic benefit and needs to be updated with effective modern solutions. One of the most promising and intensively explored therapeutic approaches against GBM is the use of nanotherapy. The first, and so far only, nanoparticle-based therapy approved for GBM treatment is NanoTherm^TM^. It is based on iron oxide nanoparticles and the thermal ablation of the tumor with a magnetic field. Numerous other types of nanotherapies are being evaluated, including polymer and lipid-based nanoformulations, nanodiscs, dendrimers, and metallic, silica, or bioderived nanoparticles, among others. The advantages of these nanoscale drug carriers include improved penetration across the blood–brain barrier, targeted drug delivery, biocompatibility, and lower systemic toxicity, while major problems with their implementation involve scaling up their production and high costs. Nevertheless, taking all the impressive benefits of nanotherapies into consideration, it seems obvious that the combined effort of the scientific world will need to be taken to tackle these challenges and implement these novel therapies into clinics, giving hope that the battle against GBM can finally be won.

## 1. Introduction

Glioblastoma multiforme (GBM) is recognized as the deadliest and most malignant primary brain tumor. It originates from astrocytic glial cells and is classified by the World Health Organization (WHO) as a grade 4 glioma [1]. It has an incidence of 1.9–9.6 cases per 100,000 people per year [2]. The incidence rises after the age of 40 and peaks in adults aged 75–84 [3], with most GBM cases being diagnosed in people in their 60s [4]. The incidence of GBM is 1.6 times higher in men than in women [4,5].

At the molecular level, GBM is characterized by the absence of isocitrate dehydrogenase (*IDH*) gene mutations (glioblastoma, *IDH*-wild type) and specific genetic alterations in the *TERT* promoter, chromosomes 7/10, and the *EGFR* gene. The presence of one of these genetic changes (e.g., *TERT* promoter mutation, *EGFR* gene amplification, the gain of chromosome 7, and the loss of chromosome 10) allows for the assignment of the highest WHO grade [6]. Furthermore, mutations in *TP53* are present in more than 60% of secondary GBM cases and 25% of primary GBM cases. Thus, defective p53 function can lead to the accumulation of genetic abnormalities, which may contribute to the formation of malignant tumors [7,8].

Therefore, GBM is characterized by a dismal prognosis and is deeply challenging for patients. With a very significant likelihood of recurrence, the median overall survival of GBM patients is only approximately 15 months [9].

Patients diagnosed with GBM generally face a low survival rate despite undergoing a combination of surgical excision, chemotherapy, and radiotherapy [10]. Even with gross total resection and combined treatment with temozolomide (TMZ), chemotherapy, and radiotherapy, most patients have a postoperative survival time of less than 2 years and a 5-year survival rate below 5%. Despite some improvements in short-term survival rates over time, the 5-year survival rate has remained largely unchanged [11,12]. It is primarily attributed to chemo- and radiotherapy resistance, leading to the imminent recurrence of GBM within the first year of therapy. Resistance and recurrence are predominantly linked to GBM stem cells (GSCs), a small subset of cells capable of regenerating tumor tissue after exposure to damaging treatments [13]. GSCs have the ability to self-renew and differentiate into various cell types, driving tumor propagation and heterogeneity and creating significant challenges for current cancer treatments. Research on GSCs is therefore crucial for developing targeted and effective anti-GBM therapies [14].

### 1.1. Current Therapeutic Approaches for GBM

Despite extensive research efforts, treatment options for GBM remain limited and often ineffective. The established protocol for treating adults under the age of 70 diagnosed with newly onset GBM, in good overall health and neurological condition, involves surgical resection, radiotherapy, and concurrent chemotherapy utilizing TMZ [15]. Therefore, whenever feasible, surgery involving gross total resection should be performed [15]. Nevertheless, the diffuse nature of GBM means that some tumor cells will almost always be left behind and continue to grow. Thus, concomitant radiotherapy is crucial as it has been shown to improve overall survival. However, traditional radiotherapy techniques expose significant volumes of normal brain tissue to radiation, increasing the risk of neurotoxicity. Efforts are underway to enable precise targeting of tumor volumes in order to minimize harm to healthy brain tissue [16,17,18].

TMZ is currently the most important drug in the standard systemic treatment of GBM [19]. At present, TMZ is the only chemotherapy drug proven to significantly improve overall survival in GBM patients and is increasingly considered for treating high-risk low-grade gliomas as well. It is an alkylating lipophilic prodrug capable of crossing the blood–brain barrier (BBB). Once TMZ reaches a physiological pH environment, it is hydrolyzed to its active metabolite, 5-(3-dimethyl-1-triazenyl) imidazole-4-carboxamide (MTIC) [20], which generates a reactive methyldiazonium ion responsible for DNA methylation. The primary cytotoxic lesion, *O6*-methylguanine (*O6*-MeG), mispairs with thymine during DNA replication, triggering mismatch repair (MMR) enzymes. MMR enzymes repeatedly remove and insert thymine, leading to persistent DNA strand breaks and eventual apoptotic cell death [19,20,21].

Despite the many clinical trials available for GBM patients, only three, i.e., the Stupp et al., EF-14, and CeTeG trials, have demonstrated positive survival outcomes in newly diagnosed, non-elderly GBM patients. In this regard, the 2005 Stupp et al. [22] trial established TMZ as the standard treatment, the 2017 EF-14 study added tumor-treating fields (TTFields) to TMZ, and the 2019 CeTeG trial combined TMZ with lomustine (CCNU) [22,23,24,25].

TTFields, a recently FDA-approved cancer treatment, have demonstrated multiple effects on cancer cells. They disrupt cancer cell division, induce DNA damage, stimulate autophagy, and activate immune responses, collectively contributing to their anticancer effects. These mechanisms work together to slow tumor growth and enhance the effectiveness of other treatments. When used alongside standard therapies, TTFields have shown promising results in improving both overall survival and progression-free survival in newly diagnosed and recurrent GBM cases [26,27]. TTFields may also enhance drug delivery in tumors with poor drug penetration due to their effects on the BBB and cell membrane permeability. The numerous ongoing clinical trials, each with a distinct approach, underscore the versatility of TTFields in various treatment protocols for both progressive and newly diagnosed GBM. This underscores the significance of this device in achieving promising outcomes while maintaining a strong safety profile [28].

Given that GBM is characterized by increased blood flow and the upregulation of vascular endothelial growth factor A (VEGF-A) and the hypoxia-induced factor (HIF), targeting VEGF-A is a reasonable approach to treatment. Bevacizumab (BEV), a humanized monoclonal antibody, was initially considered a promising treatment for GBM. It reduces angiogenesis by binding to circulating VEGF-A and disrupting its interaction with receptors on endothelial cells [29]. However, current randomized controlled trials do not provide sufficient evidence to conclusively demonstrate that BEV enhances overall survival and quality of life in patients with recurrent GBM, despite some observed benefits such as improved progression-free survival, reduced steroid use, and preserved cognitive function. Combining BEV with TTFields and administering it at the first recurrence may yield better outcomes [30]. However, external-control-based reassessments of the AVAglio and RTOG 0825 trials reaffirmed that BEV does not improve overall survival in newly diagnosed GBM [31].

### 1.2. Resistance to Standard Therapy

Despite GBM being the deadliest primary central nervous system (CNS) malignancy, its treatment protocols have remained largely unchanged for nearly two decades, underscoring the tumor’s formidable resistance to current therapies [32,33].

One of the first identified mechanisms of resistance to TMZ therapy was the increased expression of the DNA repair enzyme *O6*-Methylguanine-DNA Methyltransferase (MGMT). The methylation of *O6*-MeG caused by TMZ can be reversed by MGMT. MGMT acts by removing methyl residues from the *O6* position of guanine, counteracting the effects of TMZ. The methylation of regulatory regions within the *MGMT* gene serves as a prognostic biomarker for responsiveness to TMZ treatment. Specifically, patients with a methylated *MGMT* gene demonstrate a superior response to TMZ when combined with radiotherapy, and longer progression-free survival (PFS) and overall survival (OS) compared to those with an unmethylated *MGMT* gene [34].

Recent research indicates that MGMT overexpression is not the only factor responsible for TMZ resistance in gliomas. Additional factors contribute to TMZ resistance independently of MGMT. These include the hyperactivation of DNA repair systems, increased drug efflux, dysregulated signaling pathways, elevated anti-apoptotic activity, survival autophagy, and the presence of GSCs. Together with MGMT overexpression, these factors further enhance resistance to TMZ [35,36,37].

The absence of a functional MMR system is another significant mechanism of resistance in GBM. TMZ’s effectiveness relies heavily on a functional MMR system to convert the DNA lesions it creates into lethal damage through futile repair cycles. If the MMR system is compromised, TMZ is less effective because these critical repair attempts, which lead to DNA breaks and cell death, do not occur. The loss of MMR protein function leads to microsatellite instability, resulting in a hypermutator phenotype and often a high tumor mutation burden. This frequently causes significant molecular heterogeneity among cells, rapid tumor evolution, and the selection of tumor clones with enhanced metastatic potential and therapy resistance. Numerous studies have shown that MMR mutations can occur spontaneously or as a response to alkylating therapy in GBM [38].

**Figure 1:**
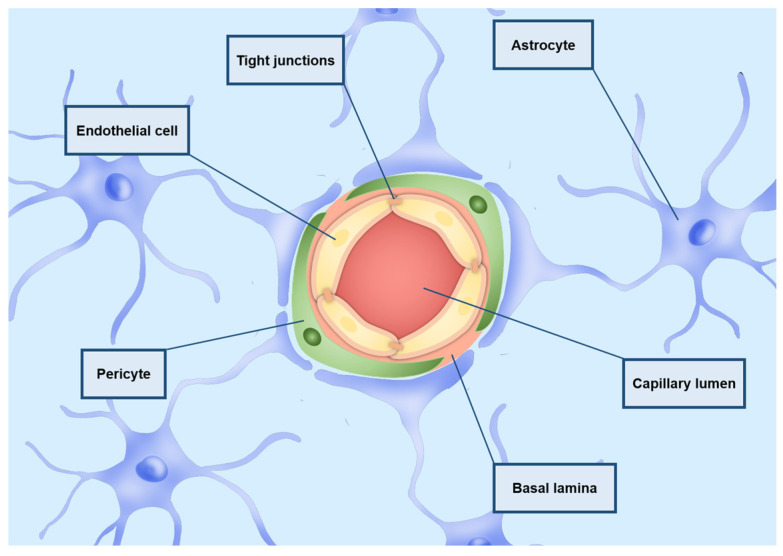
Schematic structure of BBB components.

GBM cells are also notoriously resistant to treatments due to the presence of the BBB, limiting the access of drugs to the CNS. The BBB is a semi-permeable barrier surrounding the CNS microvasculature, consisting of various cell types including endothelial cells, pericytes, astrocytes, and microglial cells. It is crucial for maintaining the brain’s unique environment, yet it poses a significant barrier to many therapeutic drugs [39]. Alongside various receptors, transporters, efflux pumps, and other cellular components, the BBB regulates the entry and exit of molecules between the bloodstream and the brain [40]. The tight junctions between brain capillary endothelial cells are primarily responsible for the BBB’s restrictive properties, severely limiting drug transfer [39]. A schematic representation of the BBB is depicted in Figure 1.

The rate at which a drug enters the CNS generally correlates with its lipid solubility. Another key factor is the molecular weight of the solute; for small molecules, when the molecular weight exceeds 400 Da, the permeability of the BBB to the drug does not increase proportionally with its lipid solubility. Lipid-soluble small molecules are thought to cross the BBB by fusing with the phospholipid bilayer [41,42].

The BBB is more stable in lower-grade gliomas than in GBM, making it difficult to treat low-grade brain tumors due to an almost intact BBB. GBM often exhibits reduced levels of claudins and occludins at tight junctions (TJs), while an increase in the VEGF results in irregular and leaky neovascularization. These vascular changes create the blood–brain tumor barrier (BBTB), characterized by higher permeability compared to the BBB. However, this increased permeability is offset by the upregulation of solute carriers and efflux transporters that remove therapeutic agents from the tumor. The breakdown in TJs mainly occurs at the tumor core, with the peripheral margins remaining resistant to macromolecule uptake [43]. Despite a disrupted BBB and more permeable vasculature, treating GBM remains challenging because the tumor is highly heterogeneous, with a necrotic core and invasive cells at the edges. Moreover, the intact BBB at the tumor edges significantly impedes effective treatment [44,45].

Various strategies are being explored to enhance drug delivery across the BBB, including both systemic and local approaches, as well as methods to increase drug penetration by modifying drugs [46,47] or the BBB itself [48,49]. High doses of chemotherapy have shown increased drug concentrations in the brain, but this approach comes with severe side effects. Overcoming the BBB could improve drug bioavailability and efficacy against brain cancers.

It is possible to bypass the BBB by adjusting the route of administration. These methods include intracerebroventricular administration (ICV), intrathecal injection, convection-enhanced delivery (CED), and intraparenchymal administration [17]. All of these methods are invasive and generate additional costs and risks for the patient. Thus, the ideal chemotherapeutic agent should be able to permeate and remain within the CNS. Researchers are investigating both invasive [50,51] and non-invasive methods such as nanoparticle-mediated drug delivery, although challenges like biotoxicity and inefficient BBB penetration persist [52].

## 2. Nanotherapy of GBM

Due to the resistance to standard chemo- and radiotherapy and the associated dismal prognosis of GBM patients, there is a necessity for novel approaches to overcome poor responsiveness to treatment. A growing body of evidence highlights the emerging role of nanotechnology in brain tumors, offering innovative and promising solutions through both nanoscale diagnostics and therapeutic methods to address these challenges [53].

Nanotechnology involves creating materials and devices at the nanometric scale. For effective permeability through the BBB, these dimensions should ideally range from 10 to 300 nm. Nanosystems can be made from various materials, and numerous nanomedicine-based CNS drug delivery approaches have been explored, including polymeric [54,55,56] and metallic nanoparticles [57,58], dendrimers [59,60], liposomes [61], lipid nanocapsules [62], and even viruses [63]. The advantage of nanodelivery systems is that they can transport numerous substances such as anticancer drugs, phytocompounds [64], or therapeutic nucleic acids (such as miRNA and siRNA [65,66]) across the BBB, enhancing their concentration in the tumor. Moreover, controlled and prolonged drug release from nanocarriers can reduce effective therapeutic doses and minimize side effects [67].

An immunosuppressive microenvironment hinders the immune system’s ability to recognize and attack cancer cells effectively. Immunotherapy has faced challenges in treating GBM, mostly because it struggles to bypass the BBB and the tumor’s immune shield [68,69,70,71]. Recent advancements have integrated nanotechnology with immunotherapy as a novel strategy to address these challenges. This innovative approach allows for the precise delivery of immunotherapeutic agents directly into the tumor microenvironment, bypassing biological barriers like the BBB. Nanoparticles (NPs), nanodiscs, and liposomes can carry drugs, antibodies, and immune cells, facilitating the delivery of combinations like immune checkpoint inhibitors alongside immunomodulators [72,73,74]. This strategy enhances drug penetration into the tumor and supports a more robust immune response, showing great promise in improving the clinical outcomes of GBM treatments. These advancements in materials science and immunology have begun to overcome long-standing technological constraints and challenges related to nanoparticle biocompatibility, marking a significant step forward in the fight against this devastating cancer.

Nanocarriers can target cancer tissue via two main methods: passive targeting and active targeting. Passive delivery involves using nanocarriers to transport therapeutics into tumors via their permeable vasculature, primarily through passive diffusion. This method exploits the leaky blood vessels in tumors, resulting from the enhanced permeability and retention (EPR) effect [75]. Solid tumors often have poor lymphatic drainage, resulting in high interstitial fluid pressure [76]. This high pressure favors the accumulation and passive penetration of nanomedicines into the tumor site, potentially enhancing the delivery and effectiveness of treatments. The size of nanopartiles significantly impacts the efficiency of the EPR effect. Small lipophilic cationic nanoparticles can passively diffuse across the cell membranes of BBB endothelial cells, although this is a rare occurrence at the BBB. Gold NPs, in particular, have demonstrated an ability to cross the BBB through passive diffusion across endothelial cells [43]. Particles smaller than 10 nm are cleared by the kidneys, while those larger than 100 nm are identified and removed by the reticuloendothelial system (RES). Therefore, the optimal size for passive targeting is between 10 and 100 nm [77]. However, passive targeting alone may not be sufficient for effective targeting [75].

On the other hand, active targeting involves modifying the drug or nanocarrier with specific ligands that bind to unique receptors that are overexpressed on target cells. This method, known as ligand-based targeting, enables nanoparticles to bind to specific biomarkers on cancer cells, enhancing drug delivery to tumors. Ligands such as proteins (antibodies), nucleic acids (aptamers), or other molecules (peptides, vitamins) are used to target these receptors, increasing the cellular uptake of nanoparticles [78,79].

Active targeting for delivering therapeutics across the BBB can leverage endogenous processes such as adsorption-mediated transcytosis (AMT), carrier-mediated transcytosis (CMT), and receptor-mediated transcytosis (RMT).

AMT involves the vectorial movement of molecules within endocytic vesicles across brain capillary endothelial cells (BCECs), from the luminal to the abluminal side where exocytosis occurs. AMT is initiated by the interaction between positively charged molecules and negatively charged microdomains on the BCECs’ surface. Unlike RMT, which relies on high-specificity binding between ligands and receptors like transferrin (Tf) or insulin receptors, AMT is driven by electrostatic interactions, resulting in lower binding affinity but higher capacity. This means AMT can accommodate a greater quantity of molecules, though with less specificity [80]. Adding cationic charges to NPs and conjugating compounds such as lectin, cardiolipin, and heparin can enhance BBB adsorption [43].

The BBB is equipped with various CMT systems that facilitate the transport of essential nutrients and metabolic substrates into the brain. Molecular biology techniques have identified several key transporters at the BBB, including GLUT1 for glucose, LAT1 for large neutral amino acids, CAT1 for cationic amino acids, MCT1 for monocarboxylic acids, and CNT2 for nucleosides [39].

The most extensively studied RMT method involves a multistage process starting with the binding of a ligand to its receptor on the brain endothelial cells’ luminal membrane, followed by receptor-mediated endocytosis, intracellular trafficking, vesicular sorting, and ultimately the fusion of vesicles with the abluminal membrane to deliver contents to the brain parenchyma [81]. The BBB surface contains numerous receptors that are highly specific for their target molecules. Utilizing nanoparticles as drug carriers in combination with compounds targeting these specific BBB receptors can facilitate the targeted delivery of hydrophobic macromolecular drugs to GBM sites [82,83]. Research has primarily focused on targets expressed on BBB cells, such as the insulin receptor (InsR), Tf receptor (TfR) [84,85,86], LDL receptor (LDLR) family members [87,88,89], melanotransferrin (MTf) [90,91], and CD98 heavy chain (CD98hc) [81].

**Figure 2:**
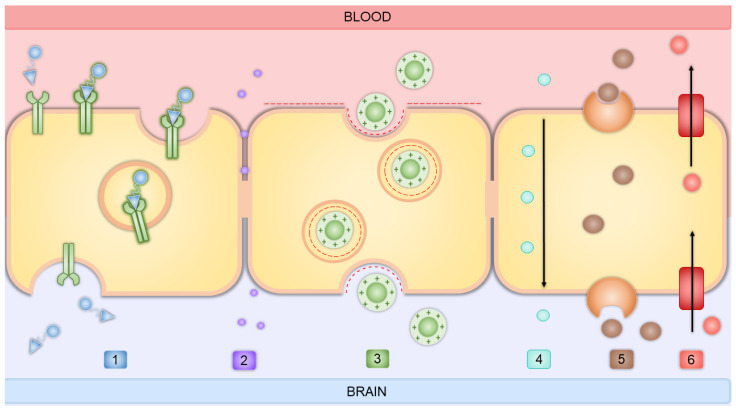
Transport across BBB. 1. Receptor-mediated transcytosis. 2. Paracellular transport. 3. Adsorption-mediated transcytosis. 4. Passive diffusion across endothelial cells. 5. Carrier-mediated transcytosis. 6. Efflux of molecules from brain to blood.

Described ways of transport through the BBB are depicted in Figure 2.

Tf is an endogenous macromolecule that transports iron ions into the brain via the TfR on endothelial cells through RMT.

LDL binds to the cell-surface domain of LDLR through apoprotein B100 (apoB-100) or apolipoprotein E (apoE), leading to endocytosis. Approximately 25% or more of the total cholesterol in the human body is located in the brain to maintain neuronal physiology. InsR on endothelial cells transports insulin from the bloodstream to the brain. InsR is still used for CNS drug delivery to the brain via RMT. TfR and LDLR-related protein 1 (LRP1) are moderately expressed in the brain, while InsR is less so. All three receptors, TfR, LDLR, and InsR, are internalized through clathrin-mediated endocytosis [92].

Effective GBM therapy often requires a two-step targeting process: first, crossing the BBB and then targeting the tumor cells. There are various cell surface markers in GBM itself, including the epidermal growth factor receptor (EGFR), erythropoietin-producing human hepatocellular (Eph) receptors, and the TfR, along with molecules such as cationic bovine serum albumin (BSA) and apoE [93]. The TfR is expressed both on the BBB and the surface of GBM cells. The significant overexpression of it in GBM, along with its reduced expression on non-tumor cells, including those surrounding the tumor, facilitates more efficient targeting and drug delivery [94].

*EGFR* is one of the most significant oncogenes in IDH-wildtype GBM. *EGFR* gene amplification is observed in 57.4% of primary GBM patients, compared to 8% of secondary GBM patients, and is linked to high levels of the EGFR protein [95,96]. EGFR is a transmembrane receptor that belongs to the human epidermal receptor (HER) family, and it is vital in various cellular processes such as growth, proliferation, apoptosis, and DNA repair. In GBM, EGFR and its ligand, the epidermal growth factor (EGF), are crucial for GBM cell invasion. Among EGFR mutations, the EGFRvIII variant is the most common, occurring in approximately 60% of cases, and it exhibits higher oncogenicity than the wild-type form (EGFR-wt) [94]. It is particularly noteworthy because it is constitutively active and serves as a potential neoantigen [95,96].

EphR are members of the tyrosine kinase receptors family. They are other cell surface proteins crucial for both the initiation and progression of adult brain cancer, making them promising therapeutic targets. Generally, Eph receptors are highly expressed during embryonic development but are downregulated or selectively expressed in normal adult tissues, making them relatively tumor-specific targets. The re-expression of EphA receptors on GSCs may reflect their role in normal development, where Eph proteins aid in niche formation and support a stem cell phenotype. It has been shown that EphA3 is highly expressed in the mesenchymal subtype of GBM. Eph receptors are frequently found on migrating tumor cells, particularly at the leading edge where GBM cells actively invade the brain parenchyma. This finding has led to the exploration of Eph monoclonal antibodies (mAbs) as potential imaging agents, which could accurately delineate tumor borders and identify areas of active invasion, potentially allowing for more complete resection and improved patient outcomes [97,98].

**Figure 3:**
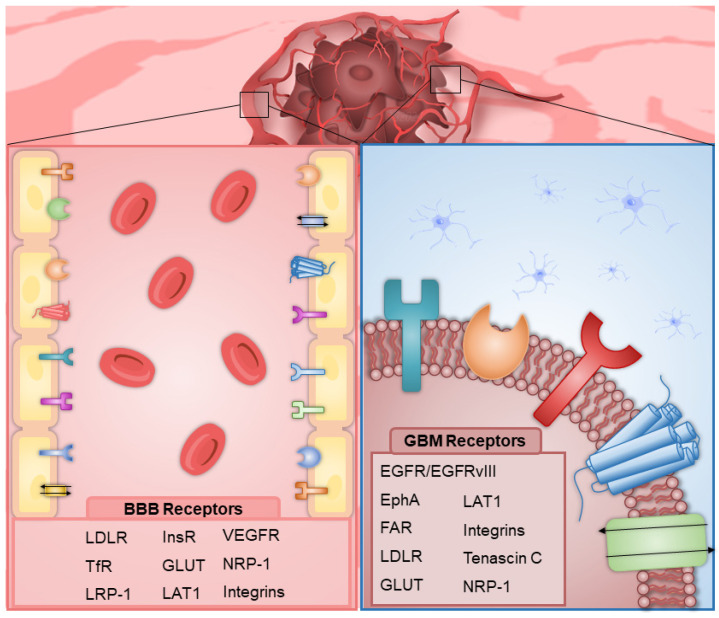
Examples of receptors present on BBB and GBM cells. BBB—blood–brain barrier, GBM—glioblastoma multiforme, LDLR—low-density lipoprotein receptor, TfR—transferrin receptor, LRP-1—LDLR-related protein 1, InsR—insulin receptor, GLUT—glucose transporter, LAT1—L-amino acid transporter, VEGFR—vascular endothelial growth factor receptor, NRP-1—neuropilin-1 protein, EGFR—endothelial growth factor receptor, EphA—erythropoietin-producing human hepatocellular receptor type A, FAR—folic acid receptor.

Folic acid is an important substrate for GBM as it plays an important role in rapidly proliferating cells by enabling DNA synthesis [99]. The demand for folic acid in these tumor cells is indicated by the overexpression of the folic acid receptor (FAR) on their surface [100]. Therefore, the combination of folic acid with NPs may increase the targeting properties of the drug delivery system [101]. Figure 3 below depicts various receptors present on the BBB and GBM cells.

Nanoparticles can also be functionalized with cell-penetrating peptides (CPPs). CPPs are short sequences of up to 40 amino acids of amphiphilic or cationic peptides which can facilitate the crossing of cell membranes and the BBB [102]. A unique feature of these peptides is their ability to be genetically or chemically modified to attach to their cargo while preserving the sequence responsible for penetration [102]. The fundamental knowledge accumulated regarding CPPs has shown their potential to enhance the efficiency of drug delivery systems by facilitating cytosolic penetration. These biologically active peptides have been demonstrated to assist in the penetration of both low-molecular-weight compounds and high-molecular-weight biopolymers. In this regard, the NFL-TBS.40–63 peptide, derived from the neurofilament low subunit (NFL), binds β-tubulin in GBM cells, altering their microtubule network and reducing proliferation. This peptide has been used to functionalize various nanocarriers, enhancing their targeting efficiency in GBM cells and other cancer cells [103,104,105,106]. Research has shown that the NFL peptide is preferentially taken up by various GBM cell lines, including human (T98G and U87-MG), rat (9L and F98), and mouse (GL261) cells, as well as human glioma-derived stem cells, inducing apoptosis while sparing healthy nervous tissue [103].

Overcoming two physiological barriers—the BBB and the tumor barrier—is pivotal for effective intravenous therapies for GBM. This challenge has led to the development of innovative nanosystems that target both the BBB and GBM, featuring multiligand surface functionalization. These nanosystems are designed to perform sequential biological functions: at first, targeting the BBB to facilitate transport from the blood to the brain, and then targeting tumor cells once they reach the brain parenchyma [107].

In parallel, nanotechnology has significantly advanced the development of intranasal drug delivery systems, enabling the targeted and efficient treatment of neurological conditions by enhancing drug absorption through the nasal mucosa. The intranasal route is particularly advantageous due to its accessibility, rich blood supply, and the reduction in first-pass metabolism, which allows for lower drug doses [108].

Nose-to-brain transport, facilitated by the olfactory and trigeminal nerves, provides a non-invasive pathway for delivering drugs directly to the CNS. The olfactory neurons, which extend from the nasal mucosa to the brain, enable this direct targeting, improving bioavailability and reducing systemic side effects [109]. However, challenges such as mucociliary clearance, enzyme-mediated metabolism, and nasal irritation must be addressed to optimize this delivery method. Advances in nanotechnology and formulation design are crucial for overcoming these obstacles and ensuring precise drug delivery to specific brain regions [110].

In the context of GBM, intranasal delivery holds promise due to its non-invasive nature and potential to target tumor sites with reduced side effects [108]. While this approach shows great potential, practical studies are limited, and challenges related to formulation stability and targeting specific brain regions remain. By developing novel nanocarriers, it is possible to enhance the efficacy and safety of nasal-to-brain drug delivery [109].

**Figure 4:**
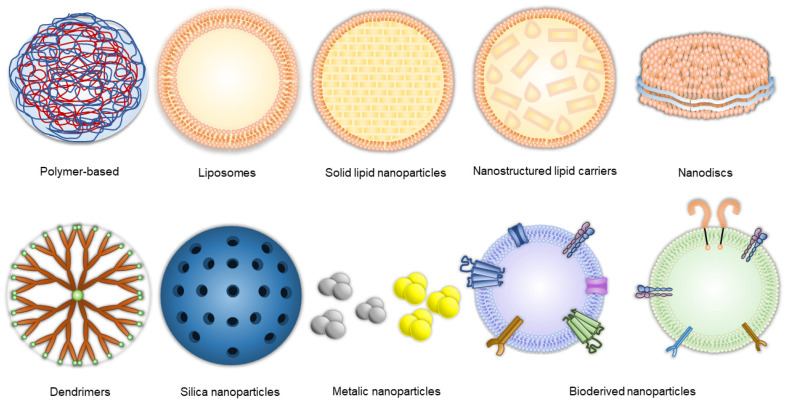
Nanoformulations currently under investigation in GBM therapy research.

Below we describe the main nanoformulations types applied in the GBM research field. Moreover, we highlight the potential of novel drug carriers like bioderived nanoparticles. Examples of nanoformulations are presented below in Figure 4.

Currently, only one nanotherapy-based treatment is being approved by both the Food and Drug Administration (FDA) and the European Medicines Agency (EMA)—it is called NanoTherm^TM^, and it was developed by a German company, MagForce Nanotechnologies [111]. NanoTherm^®^ therapy is a magnetic hyperthermia therapy (MHT), an advanced approach used in GBM patients when conventional treatments have been exhausted. This method integrates thermal ablation with nanotechnology. It utilizes a ferrofluid formulation of a colloidal suspension of amino silane coated with superparamagnetic iron oxide nanoparticles (SPIONs) suspended and distributed in 15 nm size particles [112]. This method involves the direct insertion of these iron oxide nanoparticles into the tumor or into the resection cavity wall, followed by their activation through an alternating magnetic field. This activation generates localized hyperthermia, leading to cancer cell damage and death while sparing surrounding healthy tissue. It has been shown that the treatment sensitizes GBM cells for concomitant radiotherapy or chemotherapy applications to avoid recurrences [113].

Clinical trials using MHT for GBM have shown promising results. In a phase I study, 14 patients with primary or recurrent GBM received radiotherapy with MHT, where SPIONs were injected into tumors. The treatment was safe with no major neurological side effects, and resulted in localized tumor control. Post-mortem analysis revealed that macrophages played a key role in clearing nanoparticles from the tumor [114]. A phase II study with 59 patients confirmed that MHT combined with RT significantly improved survival, and iron metabolism analysis showed that nanoparticles remained localized in the tumor without systemic release [115]. These findings led to the approval of NanoTherm^®^.

However, challenges remain. One patient developed an inflammatory response resembling an abscess, requiring surgical removal. MHT also necessitates the removal of metallic implants and pacemakers due to magnetic interference. Additionally, precise intratumoral heating is critical—excessive heat can harm healthy tissue, while insufficient heat may lead to tumor recurrence. Despite these challenges, MHT remains a promising GBM therapy, with ongoing efforts to refine its application [116]. NanoTherm^®^ represents the first, and hopefully soon not the only, nanoparticle-based therapeutic approach approved for GBM treatment. To further evaluate the efficacy and tolerance of NanoTherm^®^ therapy system in recurrent GBM, an ongoing clinical trial ANCHIALE is recruiting in Poznan, Poland (NCT06271421) [117].

### 2.1. Polymer-Based Nanoformulations

Various polymer-based nanoparticles (PNPs) are being investigated to improve delivery and deal with limited drug permeation and retention inside tumors. Polymeric materials allow for more controlled release kinetics, and increased affinity and drug concentration in targeted tissues. Modern PNPs are made from biocompatible, biodegradable materials, which often offer additional capabilities like an easily tunable surface (chitosan), safety (poly-lactic-co-glycolic acid; PLGA), and pH-dependent stability (poly-dopamine) [56,118,119].

In this context, Kuźmińska et al. [120] enclosed etoricoxib and cannabidiol in PLGA NPs, showing their ability to reduce cell viability in order to induce cell cycle arrest and the apoptosis of GBM cells. In another study, Sayiner et al. [121] developed PLGA PNP-based thermoreversible hydrogel formulations loaded with TMZ for local administration. The authors achieved a series of formulations using PLGA, polyvinyl alcohol (PVA), dimethylformamide (DMF), acetone or ethyl acetate, evaporation times, and centrifugal speeds. The A2 formulation demonstrated the highest cytotoxicity against RG2 cells at 32%, corresponding to the literature IC_50_ dose for TMZ of 100 μM.

**Figure 5:**
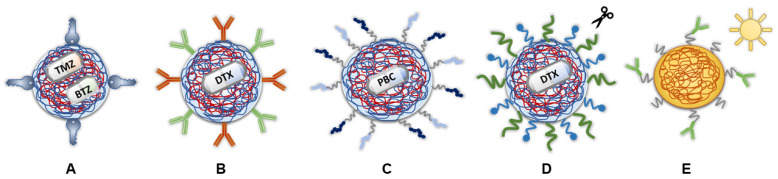
Depiction of targeted PNPs. (**A**) Tf-PLGA-PNPs loaded with TMZ and bortezomib (BTZ) [60]. (**B**) Anti-EGFR and anti-PD-L1 DTX-PLGA system [118]. (**C**) PLGA-PEG-NPs coated with T7 and R9 peptides containing palbociclib (PCB) [119]. (**D**) PLGA-PEG-Angiopep2-His formulation loaded with DTX [120]. (**E**) PPV-antiEGFRvIII fluorescent formulation [121]. Detailed description in text.

In another study, Ramalho et al. [56] evaluated the co-delivery of TMZ and bortezomib to GBM cells through PNPs made of Tf-conjugated PLGA (Figure 5A). The obtained NPs’ mean diameters were below 200 nm and had negative zeta potential, which facilitates BBB crossing and cell internalization. They were also characterized by a controlled and sustained release for 20 days. Also, a more acidic pH environment accelerated the release rate in both PLGA-PNPs and Tf-conjugated ones. Despite slower release rates, Tf-modified PNPs achieved 50% better uptake than non-modified PNPs in both U251 and T98G cell lines and 20% better uptake in the NHA cells. When using free TMZ and bortezomib, the effectiveness was lower compared to when the drugs were encapsulated in PLGA-PNPs. Specifically, PLGA-PNPs loaded with TMZ and bortezomib were much more effective in reducing cell viability in both U251 and T98G cells. Tf-conjugated PLGA-PNPs also showed improved cytotoxicity compared to free TMZ and bortezomib, though were slightly less effective than non-conjugated PLGA-PNPs. Overall, PLGA-based PNPs enhanced therapy effectiveness by almost five times compared to using the free drugs alone.

Not only have TMZ or bortezomib been tested in combination with PNPs, but also other cytostatic drugs are being evaluated in this context. Caban-Toktas et al. [119] tested a combination of PLGA PNPs loaded with paclitaxel (PTX) and R-flurbiprofen (FLUR) in an in vivo model using RG2 (rat glioma) cells to induce tumors in rats. The authors achieved PLGA PNPs, PEGylated PLGA PNPs, and chitosan-coated PLGA PNPs as emulsions with PVA or alpha-tocopherol-PEG-1000 succinate (TGPS) via the nanoprecipitation method. They loaded PNPs with PTX and FLUR. The PNP sizes ranged from 150 nm to 190 nm, assuring BBB crossing. The chitosan-coated ones were the largest and possessed positive zeta potential, while the rest of the PNPs had negative zeta potential. The control (blank PNPs and free PTX + FLUR) and loaded PNPs were administered via intraperitoneal injection on the 10th day after the implantation of the tumor cells in rats. Five days later, the rats were subjected to follow-up MRI scan measurements. The combination of PNPs with PTX + FLUR significantly reduced the tumor volume compared to the increased size in all the control, free PTX, and free PTX + FLUR groups.

In addition to inventing novel medications, it is also possible to modify existing drugs used in standard therapy and combine them with nanocarriers. The efficacy of TMZ esters was confirmed before in studies conducted by Yu et al. [118], whereas Chu et al. [122] evaluated the effectiveness of TMZ butyl ester in PLGA NPs targeting GBM via nose-to-brain delivery. Interestingly, the authors modified the evaluated NPs with the Ephrin type-A receptor 3 (EphA3) tyrosine kinase antibody to increase the cellular uptake. EphA3 is a membrane-associated receptor that can be used as a functional target for the treatment of GBM since it is overexpressed in stroma and vasculature in gliomas but not in normal tissues. A sustained in vitro release profile up to 48 h and a mean particle size of 145.9 ± 8.7 nm was reported. An in vitro cell viability assay using the 16HBE cell line confirmed the safety of these NPs for intranasal administration. The results from a rat glioma C6 cell cytotoxicity assay and subsequent experiments on specific cellular uptake demonstrated enhanced GBM targeting due to anti-EPHA3 modification. The fluorescence distribution and anti-glioma effectiveness observed in glioma-bearing rats demonstrated the effectiveness of this formulation.

TMZ hexadecyl ester (TMZ16e), another TMZ derivative, exhibits high lipophilicity, membrane permeability, and potent anti-glioma properties, showing promise in reversing drug resistance. Earlier research confirmed TMZ16e’s superior physicochemical properties and its ability to counteract TMZ resistance by lowering MGMT protein levels [123]. In this context, Wang et al. [47] created PLGA NPs modified with antibodies against EphA3 (anti-EphA3) and loaded with TMZ16e for targeted GBM therapy via intranasal administration. The results showed that these TMZ16e-loaded NPs possess suitable properties for intranasal administration. They exhibited cytotoxic effects on T98G cells in in vitro experiments and demonstrated strong anti-glioma effects in vivo in an orthotopic xenograft model. Additionally, they induced cell cycle arrest at the G2/M phase. In vivo experiments with orthotopic nude mice models indicated that intranasal delivery of TMZ16e-loaded NPs exhibited significantly reduced tumor growth. Moreover, Western blot analysis revealed that the reversal of TMZ resistance was associated with the downregulation of the MGMT protein. Overall, the authors concluded that TMZ16e-loaded NPs for intranasal delivery are effective in brain targeting, have improved anti-glioma effectiveness as compared to TMZ16e-NPs, TMZ16e, and TMZ groups, and most importantly, they extend the survival of tumor-bearing mice.

In a study of Emami et al. [124], a dual-receptor targeted strategy in preparation of PLGA NPs was evaluated. In this regard, anti-EGFR and anti-PD-L1-antibodies were used to decorate PLGA carriers loaded with docetaxel (DTX) to improve drug internalization into tumor cells (Figure 5B). The authors reported that this dual-receptor-targeted DTX-PLGA system was designed as an efficient and safe drug delivery platform. In vitro studies demonstrated that it exhibited high cytotoxicity and improved drug internalization. In contrast, systems with only one type of ligand (either anti-EGFR or anti-PD-L1) did not trigger significant cytotoxic effects. Thus, the dual-ligand approach synergistically enhanced cellular uptake and was crucial for effective DTX delivery to the tumor site.

Recently, Lo and Lin [125] developed dual-peptide PLGA-PEG-NPs containing the targeting T7-peptide and the cell-penetrating R9-peptide for the delivery of palbociclib to GBM (Figure 5C). The release of palbociclib from NPs was pH-dependent, with a faster release at a pH of 5.5 (like in endosome) compared to the physiological pH of 7.4. A faster release rate in lower pH values is desired in the case of tumor chemotherapy. The dual-peptide NPs significantly improved palbociclib transport across bEnd.3 cells and inhibited U87-MG cell growth more effectively than palbociclib alone.

Furthermore, Martins et al. [126] created multifunctional NPs for delivering DTX to GBM, aiming to target the BBB, facilitate transport to the brain, and enhance tumor cell accumulation (Figure 5D). The NPs featured a PLGA core with an acid-cleavable, long-length PEG shield linked to the Angiopep-2 peptide, which targets the LDLR at the BBB to facilitate endocytosis, and a short-length PEG shield linked to L-Histidine (His), which targets the tumor-specific LAT1 transporter to enhance BBB-to-GBM transport. The acid-cleavable nature of the NPs ensures that the long PEG-Angiopep-2 segment detaches in the acidic endosomal environment, aiding endosomal escape and efficient delivery to the brain. The multifunctional NPs also showed up to three times higher cytotoxicity in 2D and 3D models. In vivo, these NPs significantly improved survival in mice with U-87 MG tumors, outperforming non-functionalized NPs and free DTX. Adding Angiopep-2 enhanced BBB transport by over threefold, resulting in greater brain accumulation of the NPs. Martins et al. demonstrated that these stimuli-responsive, dual-ligand NPs could effectively treat GBM and may also be useful for brain metastasis and lower-grade gliomas [126].

The study of Liang et al. [127] presents the utility of a polymer other than PLGA (Figure 5E). They examined the efficacy of fluorescent a poly[2-methoxy-5-(2-ethylhexyloxy)-p-phenylenevinylene] (PPV) core conjugated with an EGFRvIII antibody. These modified NPs can specifically target GBM cells for imaging and photodynamic therapy. Fluorescent targeting could help neurosurgeons clearly identify tumor boundaries during surgery for more precise tumor removal. Additionally, the reactive oxygen species released upon light irradiation killed both EGFRvIII-positive tumor cells and nearby non-EGFRvIII-positive cells.

### 2.2. Lipid-Based Nanocarriers

Lipid nanoparticles (LNPs), including solid lipid nanoparticles (SLNs), and nanostructured lipid carriers (NLCs), have been effectively utilized in clinical trials to deliver both hydrophobic and hydrophilic drugs. These nanoparticles, capable of encapsulating therapeutic agents in aqueous or lipid-based cores, improve drug stability, reduce side effects, and target specific cells, such as cancer cells [128]. These nanocarriers have been well known for many years and are primarily composed of natural materials, making them biocompatible, biodegradable, and safe for therapeutic use [129]. They are particularly valuable in cancer therapy due to their ability to cross the BBB, a critical feature for treating brain tumors like GBM [62,130]. In addition to GBM, LNPs are also promising for drug delivery and imaging in neurological conditions like Parkinson’s disease [131,132,133], Alzheimer’s disease [134], and strokes [135,136]. Their structure enables drug-controlled release, reducing toxicity and enhancing therapeutic effectiveness. LNPs provide a stable delivery platform for targeted cancer therapy, improving the precision and effectiveness of drug delivery [137]. The primary limitation of LNPs is their tendency to fuse, especially when the average size of the synthesized formulation is below 100 nm. This fusion leads to the leakage of encapsulated substances from the lipid vesicles and enhances dispersibility. However, this issue can be addressed by applying a PEG coating to the surface of the lipid nanoparticles [138]. Ionic liquids (ILs), such as choline-2-hexenoic acid, can further enhance tissue-specific drug delivery by facilitating LNP transport across the BBB using intravenous delivery. ILs help modulate the interaction of LNPs with serum proteins, potentially increasing drug accumulation at the BBB through red blood cell hitchhiking [139]. Originally focused on liver targets, LNPs have demonstrated high encapsulation efficiencies for macromolecules such as siRNA and mRNA [140,141,142] and are now being explored for CNS applications. Enhancements like surface functionalization with targeting ligands and surfactant coatings improve brain uptake and access.

The LNP system, designed to deliver drugs and antibodies to tumor-associated myeloid cells (TAMCs), was brought to life through a meticulous formulation process. A lipid-based nanoparticle formulation, crafted with 1,2-dioleoyl-sn-glycero-3-phosphocholine (DOPC), cardiolipin, and cholesterol, forms a hydrophobic membrane with a phospholipid bilayer structure. These components create the perfect environment for the hydrophobic dinaciclib (a small molecule CDK5 inhibitor). The surface was then fortified with a DSPE-PEG2000 compound, a crucial element that stabilizes the nanoparticles, extends their circulation time in the body, and reduces their recognition by the immune system, thereby ensuring the reliable delivery of dinaciclib. The outer layer of LNPs was then functionalized with αPD-L1 by conjugating it with the terminal maleimide group of DSPE-PEG2000 to enhance their specificity in targeting cells expressing PD-L1. The modification with αPD-L1 enhances the specificity of the LNPs in targeting cells expressing PD-L1, which is beneficial for targeted drug delivery systems, particularly in cancer treatment. Cryo-EM images revealed that the spherical nanoparticles were less than 100 nm thick and were covered in monoclonal antibodies. Dynamic light scattering (DLS) showed that the αPD-L1–functionalized lipid nanoparticles (αPD-L1-LNP) were around 90 nm in diameter, slightly larger than nonmodified LNPs, and had a slightly negative charge, as indicated in the zeta-potential analysis.

The study of Razavi et al. [143] demonstrated that αPD-L1-LNPs, containing dinaciclib, effectively targeted TAMCs in vitro and in vivo in GBM. They spared other immune cells, such as T lymphocytes, which showed their high specificity. The LNPs were bound explicitly to PD-L1-expressing TAMCs and delivered dinaciclib, which led to a significant depletion of TAMCs and reduced their immunosuppressive functions within the tumor microenvironment. The combination of αPD-L1-LNPs with RT enhanced the therapeutic outcome and showcased the potential of this combination therapy. Exploiting the upregulation of PD-L1 on TAMCs post-RT improved the nanoparticle delivery and led to prolonged survival in glioma-bearing mice models. The study also validated the high targeting efficiency of αPD-L1-LNPs in human TAMCs from GBM patients, indicating the potential for clinical translation.

As a type of LNP, SLNs have advantages like effective drug delivery for both hydrophilic and lipophilic compounds, long-term stability, and reduced toxicity. However, SLNs have limitations like low drug-loading capacities and crystallization-induced drug expulsion. This restricts their use in more complex or extensive therapeutic compounds. NLCs address these limitations by incorporating liquid lipids which improve encapsulation efficiency and stability, and reduce drug expulsion during storage [144]. NLCs also utilize various surfactants to enhance their efficacy. To sum up, NLCs offer a larger drug-loading capacity while maintaining the critical advantages of lipid-based nanoparticles. Their unique ability to integrate solid and liquid lipids enhances therapeutic agents’ flexibility and storage capacity, reassuring us of their potential in challenging environments such as the brain, where the BBB often interferes with therapeutic interventions [137,139,145]. Sphingosomes, composed of sphingolipids, provide another alternative [139,145].

In the context of immunotherapy, SLNs have been intended to deliver treatments directly to TAMCs, which play a significant role in suppressing the immune system within the GBM tumor environment [143]. By attaching antibodies targeting the PD-L1 protein onto TAMCs, SLNs can precisely deliver drugs like cyclin-dependent kinase (CDK) inhibitors. These inhibitors work by depleting TAMCs, which weakens their ability to suppress the immune system.

A powerful example of this strategy is using SLNs to deliver dinaciclib encased within SLNs modified with PD-L1 antibodies. This approach not only reduces the population of TAMCs but also enhances the body’s immune response, especially when combined with radiotherapy. Radiation increases the expression of PD-L1 on TAMCs, effectively marking them as more recognizable targets for the PD-L1 antibodies carried by the SLNs. This makes the PD-L1 antibodies carried by the SLNs even more effective in homing in on and neutralizing these immunosuppressive cells [146].

NLCs were developed in response to the limited drug-loading capacity of SLNs [144]. NLCs offer a larger drug-loading capacity while maintaining the critical advantages of lipid-based nanoparticles. Moreover, their unique ability to integrate solid and liquid lipids enhances therapeutic agents’ flexibility and storage capacity [137].

Chen et al. [147] conducted a study using NLCs containing dihydroartemisinin (DHA), a compound known for its anti-tumor properties. The structure of the NLC was optimized for better stability and the controlled release of the therapeutic substance. To increase the specificity and efficacy of NLCs, they were coated with glioma cell membranes, which gave them biomimetic properties, enabling them to penetrate the BBB more efficiently and avoid recognition by the immune system. Although glioma cell membranes have some ability to target tumor cells, the study chose to further modify the carriers with the Asn-Gly-Arg peptide (NGR). This ligand binds explicitly to aminopeptidase N (CD13), expressed on the endothelial cells of newly forming blood vessels and some tumor cells. NLCs modified in this way (DHA-NGR/CCNLC) showed excellent tumor-targeting capabilities and efficient accumulation in tumor tissue, as confirmed by both in vitro and in vivo studies. Studies have assessed the efficacy of BBB and BBTB permeation and the intrinsic uptake of the formulation by glioma tumor cells. Tests in animal models showed that DHA-NGR/CCNLC significantly improved the survival time in mice with glioma compared to unmodified carriers. The nanosystems showed enhanced drug release and prolonged presence in the bloodstream, resulting in better therapeutic effects. These results suggest that NLCs, especially with appropriately selected surface modifications, may provide an effective platform for targeted GBM therapies.

The study by Song and co-authors [148] used NLCs modified with an arginine–glycine–aspartic acid peptide (RGD peptide) to deliver TMZ directly to GBM cells. The RGD peptide, known for its high binding efficiency to receptors on tumor cells and the tumor vascular endothelium, was used to increase the selectivity of drug delivery. The study developed NLCs containing TMZ, which were then coated with RGD-modified PEG-DSPE phospholipid. This innovative approach has great potential for the future of glioma treatment. A key part of the study was the optimization of the formulation, including analysis of the particle size, zeta potential, drug encapsulation efficiency, and in vitro drug release studies. The results, which showed that RGD-TMZ/NLC had significantly better stability and controlled TMZ release than unmodified carriers, provide a solid basis for further research. In vitro, RGD-TMZ/NLC showed higher cytotoxicity against U87MG glioma cells, and their IC50 value was twice as low as that of unmodified NLCs. In vivo studies with glioma-bearing mice showed that RGD-TMZ/NLC was more effective than traditional TMZ at stopping tumor growth, highlighting potential advancements in GBM treatment. The combination of NLCs and the RGD peptide significantly enhanced target specificity and therapeutic efficacy, making it an exciting strategy for the effective treatment of brain tumors, especially those resistant to conventional therapies.

In a study by Zhang et al. [149], dual-ligand-comodified NLCs (L/R-T/V-NLCs) with lactoferrin and RGD were developed for combination therapy in GBM. These carriers were loaded with TMZ and vincristine (VIN). The results showed that L/R-T/V-NLCs were stable, nanosized, and had high drug encapsulation efficiency. They demonstrated sustained drug release, high cellular uptake, significant cytotoxicity, synergy effects, increased drug accumulation in tumor tissue, and notable tumor inhibition with low systemic toxicity.

SLNs and their derivatives, like NLCs, represent a significant advancement in nanomedicine, offering targeted delivery systems capable of improving the effectiveness of immunotherapeutic agents while overcoming the biological barriers that limit traditional therapies [150,151].

In addition lipid nanoemulsions are also being evaluated. Recently, Gostyńska et al. [152] showed the antiglioma potential of a honokiol-loaded nanoemulsion. The developed formulation was characterized by good stability and a satisfactory toxicity effect on the GBM cell lines. Lipid nanoemulsions can therefore be regarded as promising pharmaceutical formulations for further development in the adjuvant therapy of GBM.

### 2.3. Liposomes

Liposomes are spherical lipid vesicles typically ranging from 50 to 500 nm in diameter. They consist of phospholipids and may include cholesterol. The consistency of liposomes allows them to self-assemble in an aqueous environment; structurally, liposomes are either unilamellar or multilamellar vesicles [153]. Liposomes can be produced using both natural (e.g., soybean phosphatidylcholine) and synthetic phospholipids. Incorporating cholesterol into liposomes is crucial as it modulates membrane permeability, alters fluidity, and enhances the stability of the bilayer membranes in biological fluids such as blood and plasma [154]. Additionally, integrating phospholipid-attached PEG into the liposome structure has proven effective in modifying liposome pharmacokinetics and biodistribution profiles [154].

Liposomes unfortunately may suffer from oxidation and hydrolysis. Chemical degradation of phospholipids can lead to alterations in lipid membrane permeability and subsequently to drug leakage. Moreover, interactions between drugs and phospholipids can also compromise the chemical stability of liposomes [155].

Recently, Piwowarczyk et al. [156] developed a cationic liposomal nanoformulation composed of two lipid types (DOTAP:POPC) for the encapsulation of four selected natural compounds (curcumin, bisdemethoxycurcumin, acteoside, and orientin) and their subsequent analysis as anti-GBM formulations. The study showed that the highest cytotoxic activity was exhibited by liposome-entrapped acteoside towards the T98G cell line with IC50 equal to 2.9 ± 0.9 µM after 24 h of incubation. In this study, a curcumin and orientin mixture in a liposomal formulation exhibited a synergistic effect against GBM. Moreover, a significant impact on the expression of apoptosis-associated proteins p53 and Caspase-3 was shown after the treatment of GBM cells with acteoside, as well as with the curcumin and orientin mixture.

Liposomes can be modified with ligands such as CPPs. The study by Mellinger et al. [104] explored the ability of liposomes functionalized with a neurofilament-derived peptide to cross the BBB and target GBM cells. The results showed the enhanced uptake of these liposomes in U-87 MG human GBM cells compared to b.End3 murine endothelial cells, indicating the peptide’s targeting capacity. The findings underscore that the NFL-TBS.40–63 peptide is a promising tool for targeting GBM after BBB passage. Future research should focus on dual-functionalized liposomes with NFL peptides and other ligands to improve BBB passage and GBM targeting in vivo.

Recent studies have also demonstrated the potential of liposomes enhanced with mApoE peptides to cross the BBB via transcytosis and deliver doxorubicin (DOX) to the brain to target GSCs. The stability of liposomes in the acidic tumor environment was confirmed, highlighting the importance of having a targeting ligand on the surface of the liposome to effectively target and release the deposited drug payload into GBM cells. The ligand is critical in targeting liposomes to the tumor site, ensuring the drug is delivered and released within the glioma cells. This optimized approach enhances treatment while minimizing off-target effects. After crossing the BBB in vitro, intact mApoE-DOX-LIPs have been shown to reduce the survival of GBM cells through the direct delivery of DOX to the cells [157]. Moreover, in GSCs exposed to radiation, there is an increase in LDLR expression, resulting in the increased internalization of mApoE/DOX/LIPs. In addition, irradiated GSCs affect the nearby BBB, increasing its permeability and inducing LDLR expression in endothelial cells. Combining radiation with liposomes in GBM therapy may thus facilitate transcytosis and improve drug delivery. In this regard, the uptake of mApoE/DOX/LIPs was increased in irradiated GSCs due to the higher expression of the LDLR. Therefore, the study provided evidence that radiation increases liposome delivery to the brain and improves tumor selectivity while protecting healthy tissues from off-target effects [157].

In study by Saha et al. [158], the liposome formulations of two novel nicotinylated amphiphiles were reported for targeting potent anticancer drugs to orthotopic mouse glioma. The use of these novel amphiphiles represents a significant advancement in the field of liposomal drug delivery. Researchers presented that the intravenous administration of the potent signal transducer and activator of transcription 3 (STAT3) inhibitor (WP-1066)-loaded liposomes in combination with in vivo dendritic cell-targeted genetic immunization is effective as an anti-GBM treatment. This approach used a DNA vaccine encoding tyrosinase-related protein-2 (TRP2). The vaccine delivered TRP2-encoding DNA, which cells then translated into the TRP2 protein to elicit an immune response. Together, these strategies showed promising results as the immunostaining of tumor sections indicated the apoptosis-inducing properties of those liposomes in GBM cells without affecting the healthy brain tissue [158]. Importantly, such targeted therapy combined with immunization markedly (>500% compared to untreated mice) enhanced the overall survival of orthotopic GBM-bearing mice. The described approach, which avoids isolating any autologous immune cells, holds significant promise for the future of GBM treatment in humans. This approach harnesses the synergistic effects of targeted chemotherapy, which directly attacks cancer cells, and in vivo dendritic cell-targeted genetic immunization, which stimulates the immune system to recognize and destroy cancer cells. In summary, these preclinical findings described herein open the door for combating GBM through this powerful combination.

**Figure 6:**
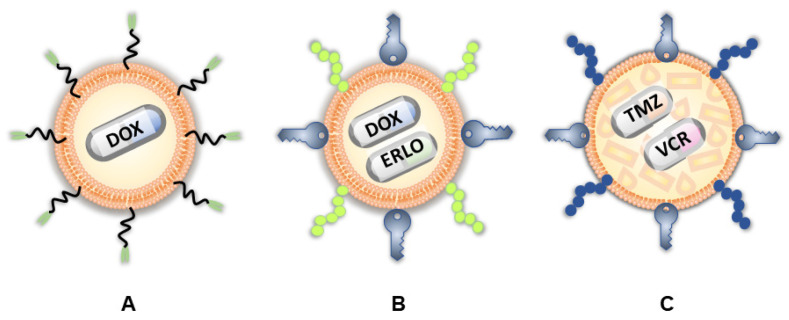
Targeted liposomes and NLC. Depiction of (**A**) EGFR-targeted immunoliposomes of Kasenda et al. [159], (**B**) Pen-Tf-functionalized liposomes of Lakkadwala et al. [160], and (**C**) Lf-RGD functionalized NLCs of Zhang et al. [149]. More details in text.

A phase I pharmacokinetic trial (NCT03603379) evaluated the tolerability and effectiveness of DOX-loaded anti-EGFR immunoliposomes for delivering drugs to relapsed GBM patients with EGFR amplification (Figure 6A). The results showed that while DOX was not detectable in the cerebrospinal fluid, its significant levels were found in the tumor tissue, indicating that the disrupted BBB in high-grade gliomas allows drug delivery to the tumor. One patient had a long remission after surgery, suggesting the potential benefits of this neoadjuvant treatment. The study concluded that anti-EGFR immunoliposomes can effectively target and deliver DOX to GBM with EGFR amplification, despite their inability to cross the intact BBB [159].

Lakkadwala et al. [160] prepared dual-functionalized liposomes and evaluated the co-delivery of DOX and erlotinib (ERLO) to treat invasive brain gliomas. These liposomes were surface-modified with Tf for receptor targeting and penetratin (Pen) for enhanced cell penetration (Figure 6B). The Pen-Tf-liposomes demonstrated excellent biocompatibility and high cellular uptake in vitro. The study showed that these liposomes efficiently crossed the BBB and delivered high concentrations of anticancer drugs to the brains of mice. Additionally, the liposomes exhibited significant anti-tumor efficacy, causing substantial regression of GBM tumors and increasing the survival time of treated mice.

### 2.4. Lipid Nanocapsules

Griveau et al. [105] created lipid nanocapsules (LNCs) functionalized with the NFL peptide. They examined the molecular structure of these nanovectors (LNC-NFL) using various microscopy techniques, including transmission electron microscopy, cryo-electron microscopy, and cryo-electron tomography. The findings revealed that the biotynylated NFL-peptide (BIOT-NFL) has the ability to spontaneously assemble to form stable, elongated filaments that strongly interact with the lipid nanocapsules, creating a structure similar to nanomolecular bracelets. This new configuration enhances internalization in rat GBM cells (F98) compared to the lipid nanocapsules alone. Overall, these results support the statement that the NFL peptide can specifically target GBM cells, both in vitro and in vivo. Furthermore, these nano-bracelets may help retain LNCs along the filaments, minimizing the off-target effects commonly associated with cancer treatments.

Researchers are also working on the improvement of the local treatment of GBM using LNCs-based hydrogels. While these hydrogels have shown promise, the LNCs released from them lack specificity for GBM cells, which could lead to off-target toxicities similar to those observed after the use of Gliadel^®^ wafers [161,162]. To address this, Gazaille et al. [163] proposed decorating the LNC surface with the NFL peptide, known for targeting GBM cells. Experiments demonstrated that NFL-coated LNCs had faster uptake by GBM cell lines (U-87 MG, F98, and GL261) compared to non-coated LNCs, while healthy human astrocytes of NHA cell line did not show increased internalization, indicating the peptide’s specificity for GBM cells. In vitro studies confirmed the improved targeting and cytotoxicity of the peptide-functionalized LNCs. In vivo studies using a murine GBM resection model showed that these functionalized LNCs localized near the resection cavity and increased survival rates. Although the hydrogels alone are not curative, they offer promising opportunities for GBM management when combined with radiotherapy and TMZ, potentially enhancing the overall treatment efficacy. This targeted delivery system could complement the Stupp protocol, improving care for GBM patients.

### 2.5. Nanodiscs

Nanodiscs are discoidal lipid bilayers, typically 8–16 nanometers in diameter, stabilized by amphipathic helical proteins known as membrane scaffold proteins (MSPs) or peptides. The lipid bilayer forms a flat, circular patch, and the MSPs wrap around its edge in a double-belt formation, shielding the hydrophobic edges from the aqueous environment. This configuration minimizes exposed hydrophobic regions, thereby enhancing the stability of the nanodisc structure [164].

High-density lipoprotein (HDL)-mimicking nanodiscs have shown promise in delivering chemotherapy and immune-boosting agents directly to the GBM microenvironment. They combine all the advantages of liposomes and nanoparticles, making them one of the most promising nanotechnology methods in GBM therapy. In fact, nanodiscs are regarded as better than other nanoparticles, such as liposomes, due to their smaller size, prolonged circulation, and improved targeting properties [165]. Evidence exists that synthetic HDL nanodiscs can cross the BBB, increase drug solubility and stability, and improve the therapeutic index by targeting the tumor site more effectively [166,167,168].

In this regard, recent studies have highlighted the therapeutic potential of sHDL nanodiscs, particularly when loaded with DTX and CpG, a TLR9 agonist, in treating GBM. In vivo experiments have shown that intratumoral administration of these nanodiscs can significantly improve median survival while minimizing widespread toxicity. Furthermore, combining DTX-sHDL-CpG with radiotherapy enhances anti-tumor effects and promotes long-term survival and immune memory in animal models. This approach effectively prevents tumor recurrence even at primary sites, where tumors recur much more frequently than at metastatic sites. It provides a potent anti-tumor response by promoting immune-mediated tumor cell death. The findings indicate that this chemo-immunotherapy approach holds solid clinical promise as a viable treatment option for patients with GBM [166].

Another study investigated sHDL nanodiscs loaded with liver-X-receptor (LXR) agonists and CpG molecules. Cholesterol is shown to be crucial in supporting tumor growth and sustaining an immunosuppressive microenvironment, thereby enabling the tumor to evade immune detection [169]. These nanodiscs address this by removing cholesterol and enhancing immune activation. All of the three tested LXR formulations exhibited similar cholesterol efflux and cytotoxicity, but one was significantly more stable. In mouse models, proper LXR formulation combined with radiotherapy yielded the highest survival rates, with long-term survivors exhibiting no tumor recurrence [170].

### 2.6. Dendrimers

Another group of NPs are dendrimers. Dendrimers are branched, highly symmetrical, globular, synthetic polymers varying in sizes [171]. They consist of a central core, branches (also called dendrons), and terminal functional groups. The branches are connected to the core and then to themselves, repeatedly resulting in an organized layered structure. One radially concentric layer created between each “level” of the branching unit is called a generation [172]. The size of dendrimers is determined by the number of generations. The cytotoxicity of dendrimers depends on the number of generations, the overall charge, and constituent dendrons [60]. The outer layer of dendrimers has a large number of exposed terminal groups, which provide hydrophilic or hydrophobic abilities. Generally, cationic dendrimers and those with a large number of generations are more cytotoxic as compared to smaller, anionic, or neutral dendrimers. Several types of dendrimers have already been evaluated in GBM research, including polyamidoamine (PAMAM), poly-L-lysine (PLL), and polypropylene imine (PPI) dendrimers. Therapeutic agents can be encapsulated within the interior cores of dendrimers or attached covalently to the surface. Overall, due to their surface area and structure, and the high functionality of branches, dendrimers are perfect carriers for a wide range of therapeutic molecules or imaging agents. Higher-generation dendrimers have greater internal space available for interactions with drugs, which improves drug solubilization; however, they have been shown to exert greater toxicity against healthy neurons compared to smaller dendrimers [60].

In order to improve BBB crossing and tumor targeting, dendrimers can be decorated with specific moieties such as hydroxyl groups, peptides, Tf, and other ligands, or they can even be camouflaged with cell membranes or coated with exosomes [173]. Research presented by Sharma et al. [174] explored the impact of PAMAM dendrimer surface modification with sugars, i.e., glucose, mannose, or galactose, to target upregulated sugar transporters in GBM cells. In this regard, D-glucose interacted with glucose transporters on tumor-associated macrophages (TAMs) and microglia, while D-galactose interacted with galactins on the surface of GBM cells, leading to the accumulation of dendrimers in the tumor microenvironment. This study shows that the modification of dendrimers with sugars moderately alters systemic biodistribution, increases cellular internalization and BBB penetration, and ensures intratumor drug delivery [174].

**Figure 7:**
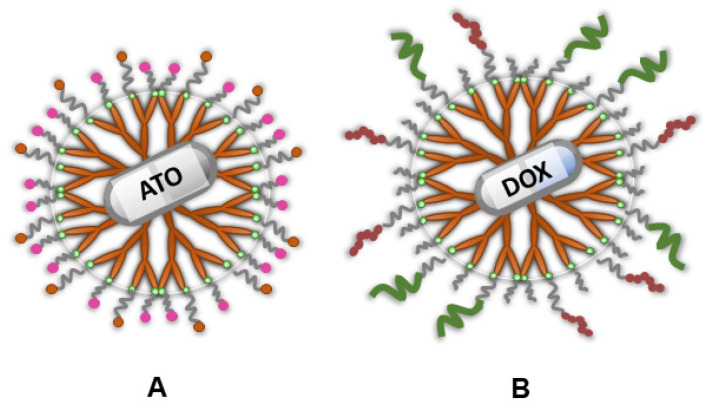
Depiction of (**A**) iRGD/TGN-PEG-PAMAM-ATO formulation by Shi et al. [175] and (**B**) EP-1-ANG2-PAMAM-DOX formulation by Liu et al. [176].

Shi et al. [175] synthesized another PAMAM (G5) dendrimer-based drug delivery system for glioma treatment. PAMAM was modified with PEG for better in vivo stability and reduced immunogenicity. Additionally, the integrin αvβ3 receptor-targeting ligand iRGD and the BBB-targeting group TGN were introduced. Arsenic trioxide (ATO) was loaded into the internal cavity through electrostatic interactions, forming iRGD/TGN-PEG-PAMAM-ATO (Figure 7A). This dual-modified system showed high entrapment efficiency (around 72%) and sustained, acid-dependent drug release. The iRGD/TGN dual-modified PAMAM with ATO significantly improved survival rates in mice. This dual-targeting approach enhanced BBB crossing, drug activation in tumor tissue, and ultimately increased therapeutic efficacy while reducing ATO side effects. This multistep-targeted delivery system holds promise for targeted glioma therapy.

Liu et al. [176] developed an EGFR-targeting peptide (EP-1) with high affinity and specificity. Then, they created a dual-targeting drug delivery system using the fourth-generation PAMAM dendrimer conjugated with EP-1 and Ang2, a peptide promoting BBB transport by binding to LRP1 on endothelial cells (Figure 7B). DOX was loaded into the dendrimer’s cavities. This dual-functionalized carrier could release the anticancer drugs in the tumor’s weak acidic environment. The combined peptides enhanced BBB penetration and glioma targeting in vitro and in vivo. The dual-targeting system significantly improved DOX’s therapeutic efficacy for glioma and reduced its systemic toxicity, showcasing a promising strategy for glioma therapy and BBB penetration.

Recent studies have shown that dendrimers are ideal for tailored medical procedures, even when they are not modified by any targeting groups. In this regard, Liaw et al. [177] found that hydroxyl-terminated PAMAM dendrimers can target neuroinflammation and glioma cells from systemic administration without the need for any targeting moieties. Dendrimer size was found to be the critical factor determining their tumor-targeting efficiency, intratumor distribution, and clearance mechanism. In this context, increasing the dendrimer size from Generation 4 to Generation 6 significantly enhanced their tumor accumulation (~10-fold greater at 24 h), tumor specificity (~2–3-fold higher), and tumor retention. This effect was associated with the reduced renal clearance rate of Generation 6 dendrimers, resulting in a longer circulation time as compared to G4 dendrimers.

### 2.7. Metalic Nanoparticles

Metal-based nanoparticles are among the most promising tools for cancer therapy. Recent research in this field has focused on gold nanoparticles, iron oxide nanoparticles (IONPs), and SPIONs. These nanoparticles can cross the BBB and effectively deliver drugs and other therapeutic compounds, including nucleic acids like microRNA, to the tumor site. Metal-based NPs have been shown to not only slow the progression of GBM but also reduce the radioresistance of cancer cells [178].

#### 2.7.1. Gold Nanoparticles

Studies show that combining biotinylated NFL peptide (BIOT-NFL-peptide) and gold nanoparticles can lead to the formation of targeted BIOT-NFL-peptide nanofibers decorated with gold nanoparticles. Researchers observed the internalization of these gold nanoparticles when coupled with BIOT-NFL nanofibers in F98 rat GBM cells. They showed enhanced uptake compared to the control group with non-coupled gold nanoparticles. This study highlighted the significant interaction between BIOT-NFL nanofibers and gold nanoparticles, resulting in increased uptake by GBM cells when treated with this combined system. Overall, BIOT-NFL-peptide shows potential as a promising therapeutic agent for targeting GBM, inspired by the interactions between peptide nanofibers and various nanocarriers [103].

A new method has been developed to synthesize gold nanoparticles combined with BIOT-NFL-peptide. This approach, termed “Method IN”, facilitates specific interactions between the BIOT-NFL-peptide, polyethylene glycol diacid (PEG-COOH), and gold salt (Au III), resulting in multifunctional hybrid nanocarriers known as BIOT-NFL-PEG-AuNPs. In a study conducted by Griveau et al. [106], researchers expanded the strategy to include peptides like TAT and Vim, focusing on the ex vivo effects of gold nanoparticles composed of BIOT-CPP-peptides (NFL, TAT, or Vim) combined with the biocompatible polymer PEG-(COOH)_2_ on rat GBM cells (F98). They assessed the efficacy of PEG-AuNPs alone compared to the BIOT-CPP-peptides in terms of mitochondrial activity and their internalization into F98 cells. The results confirm the feasibility of synthesizing new multifunctional nanovectors made from BIOT-CPP-peptides and PEG-AuNPs. Three BIOT-CPP-peptides were tested on rat GBM cells, demonstrating improved cellular internalization when combined with CPP-peptides, along with their localization within organelles and effects on mitochondrial function.

In another study, Arib et al. [179] combined PEG molecules and BIOT-CPP-peptides (BIOT-NFL, BIOT-VIM, or BIOT-TAT). The peptides are capable of stabilizing gold nanoparticles (AuNPs) through complexation between their ketone and amino groups and chloride auric ions. By utilizing biotinylated CPP (BIOT-CPP) complexes with gold ions, they created stable nanoparticles (BIOT-CPP-PEG-AuNPs) with theranostic (combining therapy and diagnostic) potential. The cellular uptake was evaluated in pancreatic (PDAC; MIA PACA-2) and GBM F98 cancer cells. The photothermal effect of the NPs was observed at a concentration of 100 mmol/L. In the presence of the peptide, a similar response was observed with or without irradiation. For PEG-AuNPs without the peptide, a 60% decrease in cell viability was noted at 24 and 48 h post-internalization. However, for BIOT-NFL-PEG-AuNPs, there was a significant reduction in cell viability: around 60% after 5 min and 45% after 10 min of irradiation, with viability at approximately 40% and 30% after 48 h for 5 and 10 min of irradiation, respectively. The authors also assessed the chemical stability over several months, biological interactions, and penetration in pancreatic (PDAC, MIA PACA-2) and GBM F98 cancer cells. This study demonstrated that BIOT-CPP-PEG-AuNPs effectively penetrated cancer cells and, upon irradiation, significantly reduced cell viability. These findings highlight the potential of this nanoformulation for phototherapy applications by improving targeted cytotoxic effects while maintaining chemical stability over time.

#### 2.7.2. Iron Nanoparticles

Yao et al. [180] developed a nanoparticle system consisting of chlorin e6 (Ce6)-conjugated iron oxide (Fe_3_O_4_) nanoparticles for the in vitro ablation of GBM cells by combining photothermal therapy (PTT) with photodynamic therapy (PDT). These Fe_3_O_4_-Ce6 nanoparticles were synthesized by conjugating Ce6 to Fe_3_O_4_ nanoparticles. The effects of this combination were evaluated on C6 cells and bEnd.3 cells. The Fe_3_O_4_-Ce6 nanoparticles acted as photothermal agents and photosensitizers, mediating PTT and PDT by producing heat and reactive oxygen species (ROS) under 808 nm and 660 nm laser irradiation, respectively. This treatment significantly reduced cell viability and killed cancer cells, as verified by CCK-8 analysis and live/dead staining. Fluorescence imaging confirmed ROS generation inside the cancer cells treated with Fe_3_O_4_-Ce6 nanoparticles and laser irradiation. Due to their excellent fluorescence properties, Fe_3_O_4_-Ce6 nanoparticles hold potential for fluorescence imaging-guided combination therapy for cancer.

IONPs have reached clinical stages due to their safety and biocompatibility. Chen et al. [181] synthesized IONP@PTX, which showed good stability and effective PTX loading and release. Researchers found that while both IONP@PTX and PTX inhibit U251 glioma cells, IONP@PTX is less toxic to HMC3 cells, indicating reduced toxicity. IONP@PTX was more effective than PTX alone in reducing glioma cell viability, migration, and invasion, linked to autophagy activation. IONP@PTX also induces ferroptosis, a cell death mechanism characterized by lipid peroxide accumulation. It increases iron ion concentration, ROS, and lipid peroxidation, and down-regulates the GPX4 protein. The autophagy pathway enhances these effects. In vivo, IONP@PTX significantly reduced tumor volume in GBM xenografts without obvious toxicity. The effects were modulated by autophagy regulators. These results suggest that IONP@PTX suppresses tumor growth through autophagy-dependent ferroptosis and may be a promising agent for tumor therapy.

The study by Fernández-Acosta et al. [182] presented evidence for inducing ferroptosis in cancer cells using novel iron oxide nanoparticles (IONP–GA/PAA). These nanoparticles, coated with polyacrylic acid (PAA) and gallic acid (GA), exhibit stability, biocompatibility, and superparamagnetic behavior, making them suitable for drug delivery and biomedical uses. IONP–GA/PAA induced 100% cell death after 24 h in HT1080 (fibrosarcoma) cells and a noticeable reduction in viability in U87MG and U373MG (GBM cell lines). However, for U87MG, U37MG, IMR32 (high-risk MYCN amplified neuroblastoma), and HT22 (neuronal, non-tumorigenic) cell lines, the 100% cell viability was achieved after 48 h. These results highlight the need for in vivo selectivity studies. It is crucial to note that although IONP–GA/PAA demonstrates significant toxicity towards the HT22 cell line, its selectivity can only be accurately evaluated through in vivo experiments. Research indicates that two key molecular factors make cancer cells more susceptible to ferroptosis induced by IONP: EPR and the increased iron dependency of cancer cells. Overall, IONP–GA/PAA nanoparticles induce ferroptosis, presenting a new approach to cancer therapy. Further research is needed to optimize their therapeutic potential and elucidate precise mechanisms. However, while 100% cell death may demonstrate potent cytotoxicity, it raises questions about selectivity, therapeutic relevance, and reproducibility that warrant careful investigation before considering this treatment for clinical applications.

A study by Chang et al. [183] focused on developing low molecular weight, hyaluronan-oleic, acid-coated, superparamagnetic iron oxide nanoparticles (LMWHA-SPIONs). The SPIONs, coated with oleic acid for colloidal stability, exhibited minimal aggregation and particle sizes mainly between 4 and 10 nm, confirming their superparamagnetic properties suitable for magnetic resonance imaging (MRI). The researchers demonstrated that LMWHA-SPIONs specifically targeted U87MG GBM cells, accumulating more iron than in normal fibroblasts. LMWHA-SPIONs displayed significant cytotoxicity toward U87MG cells while remaining harmless to NIH3T3 fibroblasts, attributed to the higher expression of CD44 receptors on cancer cells. Overall, the findings suggest that LMWHA-SPIONs hold promise for targeted cancer therapy and diagnostic imaging, offering tumor specificity with reduced impact on normal cells.

### 2.8. Silica Nanoparticles

Silica-based nanoparticles (SNPs) have gained significant attention as drug-delivery vehicles due to their distinct structural features, such as their high surface area, tunable porosity, and excellent biocompatibility [93]. These nanoparticles offer great potential for therapeutic applications, particularly in oncology, having a porosity of up to 80% that enables high drug loading and adjustable dimensions, allowing for precise delivery to target tissues [93]. Research has shown that smaller SNPs, particularly those under 50 nm, are more effective in delivering drugs across biological barriers like the BBB [184]. Developing ultra-small silica nanoparticles with enhanced pore sizes has become the primary concern in excelling SNP advancement.

Janjua et al. [185] took the challenge of decreasing the size while increasing the porosity of SNPs, achieving ultrasmall (30 nm), large-pore (7 nm) SNPs. The developed particles were then PEGylated and loaded with TMZ according to the methods developed by their team. The last step involved the attachment of lactoferrin molecules onto the surface of the loaded SNPs, yielding PEGylated loaded SNPs (PEG-SNPs) and SNPs conjugated with lactoferrin (Lf-PEG-SNPs). The developed conjugates were tested for in vitro BBB permeation and uptake in a model made of immortalized human cerebrovascular endothelial cells hCMEC/D3, which mimic the tight monolayer of the physiological BBB. The developed systems were rapidly uptaken by endothelial cells via transcytosis in a concentration-dependent manner—the higher the concentration, the higher the uptake rate. The results also suggested that PEG-SNPs can influence the integrity of tight cell junctions, while the Lf-PEG-SNPs are prone to be uptaken via receptor-mediated endocytosis. The in vitro uptake studies revealed that the PEG-SNPs demonstrated superior performance, resulting in similar uptake with higher accumulation in the U87 cells. However, the Lf-PEG-SNP exhibited the highest efficiency in the cytotoxicity assay, causing the most considerable number of cells to undergo apoptosis, compared to the PEG-SNPs and TMZ alone.

Luo et al. [93] obtained another targeted SNP–Tf-conjugated porous SNPs (Tf-SNPs) loaded with DOX. The Tf-SNPs demonstrated the highest affinity towards U87 cells compared to HaCat and hCEMC/D3 cells. Moreover, Tf-SNP demonstrated a favorable pH-dependent releasing profile, having the highest release rate in a pH of 5. Cytotoxicity tests revealed that free DOX killed 34.6% of the U87 cells as compared to the considerably more efficient Tf-SNPs loaded with DOX which killed 75.1% of the cultured cells. This study showed that the active efflux and passive diffusion of the drug from the cell could be counteracted, while favorable kinetics of release and increased internalization of the drug-loaded particles can be achieved thanks to the Tf-SNP platform.

Zhang et al. [186] took a different approach to functionalizing commercially available mesoporous silica nanoparticles (MSNs). The MSNs were coated using polydopamine (PDA), a dopamine-derived synthetic melanin polymer characterized by its biosafety, biocompatibility, and pH-reliant stability, which provides controlled release capabilities. After coating, Zhang introduced additional RGD functionalization of the MSN, yielding MSN-RGD. The RGD ligand exploits the presence of integrin receptors on the surface of tumor cells, thus improving targeting and decreasing their migration. The obtained MSN-RGD were then loaded with TMZ and/or Chloroquine (CQ), which is an autophagy inhibitor, and underwent release studies. Drug-loaded MSN-RGD performed best in the 5.0 pH buffer, yielding 96.61% release; however, at the physiological pH of 7.4, the release ratio was still high, ranging up to 55.97%. The in vitro tests on the U87 cell line resulted in MSN-RGD loaded with both CQ and TMZ, inducing the highest apoptosis of 58.26% viable cells, while the TMZ MSN-RGD induced apoptosis in 38.24%. Free TMZ induced apoptosis only in 20.41% of cells. The obtained systems were also tested in the in vivo models of murine GBM. The study included 24 male mice with induced tumors. The combination of TMZ/CQ@MSN-RGD was the most effective in tumor growth rate inhibition and caused the most considerable decrease in tumor volume. Moreover, post mortem tissue analysis proved that the loaded MSN-RGD systems were not significantly toxic and maintained a high affinity for malignancy observed in the in vitro studies. Overall, the study showed that the drug-loaded MSN-RGD had low toxic potency, reduced chemotherapy resistance, and inhibited the proliferation of glioma cells.

### 2.9. Bioderived Nanoparticles

#### 2.9.1. Exosomes

One of the major concerns of synthetic and inorganic nanoparticles is their biocompatibility and their ability to be safely excreted or degraded within the patient’s body. In recent years, scientists have been trying to develop bioderived nanocarriers for GBM treatment. As opposed to artificial nanocarriers, biologically derived ones have many advantages including lower immune recognition, low toxicity, and the ability to penetrate stringent biological barriers, such as the BBB. In this context, exosomes, naturally occurring nanovesicles involved in intercellular communication, have emerged as a novel and promising approach for delivering therapeutic agents to tumors of the brain. This innovative method has garnered significant scientific interest, with ongoing research exploring its potential to overcome the limitations of traditional drug delivery strategies. The recent literature highlights their application in efficient drug delivery to GBM cells [187,188,189,190,191].

Exosomes are small extracellular vesicles (EVs) with a lipid bilayer membrane, secreted by almost all mammalian cells. They play a crucial role in cell–cell communication, both in normal physiological processes and pathological conditions like cancer. They have a nano-sized diameter of 30 to 100 nm. Although priorly thought to be cellular “trashbags”, exosomes serve an essential role as natural mediators, transporting a variety of biomolecules, such as proteins and ribonucleic acids (RNAs), from parent cells to recipient cells. They derive from cellular structures, thus making them uniquely biocompatible. Exosomes also possess the ability to deliver hydrophilic and hydrophobic molecules, which has made them promising candidates for drug delivery, particularly in cancer therapy and diagnosis [192,193]. The literature also reports the intrinsic tissue and cell-targeting properties conferred by surface structures such as tetraspanins and integrins [187]. Despite their advantages, ongoing research aims to optimize exosome-based therapies by engineering their surface molecules to enhance targeting capabilities and identify the most suitable cell types for exosome derivation.

Zhu et al. [193] developed embryonic stem cells-derived exosomes (ESC-exos) with improved tumor targeting by modifying the surface of the vesicles with the Cyclo (Arg-Gly-Asp-D-Tyr-Lys) peptide (c(RGDyk), obtaining cRGD-ESC-exos. The study showed that ESC-exos alone had antiproliferative activity towards U87 and U251 cell lines. Moreover, the ESC-exos were loaded with PTX yielding PTX-EXO, and subsequently, the peptide was conjugated following the postinsertion method, yielding cRGD-EXO-PTX. The cRGD-EXO-PTX demonstrated higher fluorescence compared to EXO-PTX in the in vivo model study, suggesting better targeting abilities of cRGD-EXO-PTX. The cytotoxicity was studied in the orthotopic murine brain tumor model. The control, PTX, cRGD-EXOs, PTX-EXO, and cRGD-EXO-PTX were administrated via the caudal vein every second day for 4 weeks. In the cRGD-EXO-PTX group, Zhu observed the strong inhibition of growth and the most considerable decrease in the size of the tumors. The group administered with PTX-EXO also exhibited less inhibition of tumor growth. However, almost 40% (compared to 20% from PTX-EXO) of the viable cells from the cRGD-EXO-PTX group were Tunel-positive, which indicated higher effectiveness of the targeted approach in bringing cells into the apoptotic state.

In a different study, Araujo-Abad et al. [192] tested GBM-derived small EVs loaded with TMZ or EPZ015666 (arginine methyltransferase-5 (PRMT5) inhibitor). The EVs were derived from primary cell lines obtained from seven GBM patients and were characterized for the presence of cell proteins. The proteins that were typical for EVs were TSG101, Alix, and CD63. Interestingly, the mean diameters of EVs depended on the cell line from which they were obtained. The drugs were loaded into EVs using two methods. The first one consisted of the treatment of the cells for at least 48 h with high doses of a drug, followed by the isolation of small EVs from the medium, while in the second method, called “direct incubation”, small EVs were first isolated from the cell line and then incubated in a medium containing a low dosage of the drug. According to the HPLC analysis, the second method turned out to be more effective in drug loading. In the cytotoxicity tests TMZ-EVs exhibited a subtle reduction in cancer cell proliferation, which was slightly lower than in the case of the free TMZ. However, the authors used very low concentrations of TMZ, from 1.25 μM to 12.5 μM, and corresponding formulations of the EVs. Similar outcomes were achieved with the EVs loaded with EPZ015666, although the reduction in proliferation was even smaller than it was for TMZ. Nevertheless, the reduction in the total amount of drugs needed to trigger an effect on tumor cells was observed in this study.

#### 2.9.2. Macrophage Cell Membrane-Based Nanoparticles

The innate immune system’s initial response to infection involves various immune cells, particularly macrophages, which play a crucial role in targeting pathogens. Macrophages exhibit active targeting capabilities, high immune compatibility, and prolonged circulation times [194]. Traditionally, they are divided into classically activated M1 macrophages and alternatively activated M2 macrophages, distinguished by differences in surface receptor expression, secretory profiles, and functional roles. However, studies on in vivo wound healing suggest that macrophages initially exhibit a pro-inflammatory M1-like response during the early stages but later transition to an anti-inflammatory M2-like state as healing progresses. Macrophage polarization refers to the activation state of a macrophage at a given moment, but due to their plasticity, this state is not permanent and can shift in response to signals from the microenvironment, such as cytokines, growth factors, inflammation, infection, injury, and hypoxia. Although macrophage polarization is often described as a fixed classification, M1-type macrophages can shift to an M2 phenotype and vice versa depending on external stimuli. Rather than a simple M1 vs. M2 binary system, macrophage polarization exists as a spectrum of activation states, where these two subtypes represent the extreme ends [195]. By leveraging macrophages’ properties, macrophage membrane-coated nanoparticles can effectively evade immune clearance while targeting tumors and inflamed tissues, addressing challenges related to biocompatibility, short circulation times, and immunogenicity often faced by traditional materials [194].

These macrophage membrane-coated nanoparticles offer a promising platform for drug delivery and therapy, enhancing drug retention at inflamed or tumor sites and neutralizing endotoxins to ensure safe and effective treatments. Their potential applications include cancer, immune diseases, atherosclerosis, infections, and inflammation, making them attractive candidates for clinical trials and biomedical applications due to their immune compatibility and targeting capabilities. Macrophage membranes, in particular, are effective for tumor targeting because they possess genuine membrane proteins that allow them to adhere to vascular adhesion molecules on cancer cells [196].

Macrophages, which are abundant in tumor microenvironments and play roles in tumor progression and metastasis, can bind to metastatic cancer cells, aiding their survival and growth. [197].

M1 macrophages are effective against tumors due to their production of pro-inflammatory cytokines and ability to internalize particles, ensuring effective drug delivery. In contrast, M2 macrophages, also known as TAMs, often promote tumor growth. In a recent study, researchers developed a drug delivery system using M1 macrophages loaded with DOX-loaded PLGA nanoparticles, which protects the macrophages from DOX toxicity while maintaining their targeting capabilities. This system demonstrated efficient tumor infiltration and showed promising results in preclinical models, prolonging survival and minimizing cardiotoxicity, thus offering a targeted approach for glioma therapy [198].

In another study, Cao et al. [199] developed nanoparticles camouflaged with macrophage membranes to evade the immune system and target GBM cells more effectively. The mesoporous polydopamine (MPDA) nanoparticles with Angiopep-2-modified macrophage membranes were loaded with small activating RNA (saRNA) to boost the expression of ALOX15, a gene critical for inducing ferroptosis. The modified nanoparticles could cross the BBB and reduce immune clearance. Once inside the tumor cells, they cause mitochondrial dysfunction, leading to cell death. In animal models, these NPs not only inhibited tumor progression but also increased the effectiveness of radiation therapy, suggesting a promising new strategy for GBM treatment by combining biomimetic techniques and ferroptosis induction.

Wu et al. [191] developed NPs by encapsulating catalase (CAT) into SNPs (CAT@SiO_2_), making a biodegradable platform termed CSI. Then, it was loaded with the sonosensitizer indocyanine green (ICG). The nanoplatform CSI was further coated with AS1411 aptamer-modified macrophage exosomes to form CSI@Ex-A. Macrophage exosome membrane coating was used to evade the immune system and target tumor cells more effectively. In GBM cells, high levels of GSH prompted the degradation of CSI@Ex-A, releasing CAT that converted H_2_O_2_ into O_2_, thus alleviating the tumor’s hypoxic microenvironment. Remarkably, CSI@Ex-A effectively curbed GBM tumor metastasis, likely by mitigating hypoxia and inhibiting HIF-1α activation. Additionally, the depletion of GSH and the self-supply of O_2_ significantly improved the efficiency of sonodynamic therapy (SDT). By disguising the nanoparticles as macrophage exosomes, they can specifically target GBM cells, as macrophages naturally hone in on tumor sites. The biodegradable nature of these NPs ensures they break down into harmless substances after delivering their therapeutic payload, reducing potential side effects. Preclinical studies have shown that this approach significantly enhances the effectiveness of SDT, offering a more targeted and efficient treatment for GBM while minimizing damage to healthy tissue.

#### 2.9.3. Virus-like Particles

Besides liposomes, dendrimers, micelles, inorganic nanoparticles, and virus-like particles (VLPs) based on the hepatitis B core antigen (HBc) or the tobacco mosaic virus (TMV), have emerged as promising drug nanocarriers [200,201]. VLPs offer several advantages, including protection against drug degradation, improved targeting to specific sites, and excellent biocompatibility and biodegradability. Unlike synthetic delivery vehicles, VLPs are homogenous in size, cost-effective to produce via recombinant expression, and demonstrate minimal toxicity. Furthermore, they self-assemble into hollow, noninfectious structures, making them safe for therapeutic use [201]. Another key feature of VLPs is their ability to be site-selectively modified, allowing precise control over drug loading, which is critical for enhancing cancer targeting and enhancing delivery. For instance, HBc VLPs can be functionalized with peptides like (TGNYKALHPHNG [TGN]) to increase the BBB crossing rate and brain accumulation, or RGD to increase their targeting specificity towards GBM [201]. Additionally, the morphology of VLPs plays an important role in their treatment efficacy, as GBM-associated vasculature contains nanodimensional pores that may affect nanoparticle extravasation. The nanoscale size of VLPs (below the 200 nm threshold) allows them to navigate these pores easily.

Finbloom et al. [200] tested three different VLPs for convection-enhanced delivery of DOX for treating intracranial tumors in U87-Luc mice. Each of the tested VLPs had a distinctive shape: MS2—spheres, Nanophage (NPh)—filament rods, and TMV—disks. VLPs-DOX conjugates were PEGylated to improve their biodistribution and combat hydrophobicity. Prior to testing on mice, VLPs were tested for cytotoxicity against U87-MG cells, which resulted in a similar dose-dependent effect of a reduction in cell viability. In the cellular uptake test, the MS2-DOX and TMV-DOX presented similar fluorescence, while the NP-DOX exhibited lower cell fluorescence, indicating lower cellular uptake of this VLP. In the in vivo test, the mice were divided into two cohorts of small-volume tumors and large-volume tumors. The small tumor treatment groups of TMV-DOX and MS2-DOX responded significantly better than the large tumor groups. Furthermore, the MS2–DOX and TMV–DOX treatments significantly increased the survival time of mice as compared to the PBS and DOX control groups. The TMV-DOX group had the longest survival time, as three out of eight mice exceeded 40 days survival time, which was not achieved for any other group.

Also, Yang et al. [201] developed a VLP based on the core of the hepatitis B (HB) virus, with the addition of TGN and RGD peptides. As chemotherapeutics, they chose to package VLP with PTX and the Yes-associated protein (YAP) siRNA. The proposed siRNA is a short nucleic acid chain that interferes with the YAP, a commonly overexpressed co-activator of the Hippo Pathway [202]. The TGN/RGD-HB VLPs were obtained via the expression of wild-type HB particles and the RGD-modified HB particles by *Escherichia coli*. The wild-type HB particles were then conjugated with TGN peptide. Both modified HB particles were then disassembled, mixed, and reassembled together to form TGN/RGD VLPs. The encapsulation process of the siRNA was conducted in wild-type HB, eventually yielding siRNA@TGN/RGD-VLPs. The PTX loading was conducted in the modified HB, resulting in PTX@TGN/RGD-VLP formation. The cytotoxicity against U87 cells indicated that PTX/siRNA@TGN/RGD-VLPs was the most effective treatment across all tested groups in all concentrations. Furthermore, PTX@TGN/RGD-VLPs also exhibited improved performance compared to the free PTX and free PTX/siRNA, but only in the highest tested concentration. The effects of developed VLPs were also tested in U87-Luci tumor-bearing nude mice. The accumulation and retention in the brain after intravenous administration were the highest in the TGN/RGD-VLPs group compared to modified HB VLPs. The results from the U87-bearing mice demonstrated that treatments with PTX/siRNA@TGN/RGD-VLPs resulted in evident regression of tumor growth. Also, the median survival time was the highest for the PTX/siRNA@TGN/RGD-VLPs (23 days), compared to the saline control group (15.5 days) and PTX@TGN/RGD-VLPs (20 days). This proved the positive additive effect of the PTX/siRNA combination with TGN/RGD-VLPs.

## 3. Discussion

In this manuscript, we presented the updated scientific data regarding nanotechnology applications in the treatment of GBM. Nanotherapy offers potential for GBM treatment but faces significant challenges. Ensuring the safety of nanomaterials remains crucial. Before clinical use, NPs must be proven non-toxic, as their size, shape, and other properties can influence their toxicity. Their small size results in a high surface-to-volume ratio, which can lead to rapid cargo release and potential toxicity. While organic nanomaterials like liposomes have been well studied with some approved for clinical use, there is still a lack of long-term safety data for inorganic nanomaterials [83]. Long-term toxicity and side effects are a serious concern, with potential unintended interactions with healthy tissues and uncertain biocompatibility [203]. The field of nanotoxicology assesses nanoparticle toxicity, but clinical translation and commercialization face challenges due to inconsistent drug release profiles and batch variability. Regulatory approval is complicated by the lack of specific guidelines, leading to prolonged evaluations. Comprehensive regulatory guidelines are needed to streamline the approval process for cancer nanotherapeutics.

Effective drug delivery to the brain requires overcoming the BBB, which restricts drug entry due to its physical, transportation, metabolic, and immune barriers. Nanocarrier design can help by reducing enzymatic reactions and immune clearance, improving drug stability.

Understanding cancer cell physiology, the tumor microenvironment, and drug-carrier pharmacokinetics is crucial for developing successful nanotherapeutics [199]. Moreover, the individualized nature of cancer requires adaptable NPs for personalized treatment, which is complex and costly, hindering large-scale production. Ensuring the penetration depth of NPs is also challenging, complicating their clinical application. Additionally, regulatory guidelines for nanotechnology are still developing, and ethical considerations need to be addressed [197].

Tumor heterogeneity complicates treatment outcomes, as different tumor areas may respond differently. NPs often accumulate unevenly within tumors due to the abnormal tumor microenvironment. The EPR effect, while helpful for targeting, can hinder NPs uptake and distribution.

Despite the potential of nanodrug delivery systems (NDDSs) to enhance GBM treatment, few have reached clinical trials. Combining NDDSs with other therapies, like immunotherapy, shows promise [204].

Future research should focus on optimizing nanoparticle design, improving targeting, and refining drug release mechanisms to enhance clinical efficacy. Continued innovation and well-designed clinical trials are crucial for advancing NDDSs in GBM therapy, aiming for more effective treatments with fewer side effects [205]. For example, combining TMZ and DOX in PEGylated liposomes showed median overall survival rates of 17.6 and 13.4 months in phase II trials (NCT03603379). PEG-DOX with continuous TMZ and radiotherapy achieved a 30.2% progression-free survival rate after one year, but overall outcomes remained modest, likely due to drug resistance or insufficient therapeutic activity [206]. Combination therapies and advanced nanomaterials like gold and magnetic iron-oxide nanoparticles show promise for future GBM diagnosis and treatment, but further research and biosafety evaluations are needed [205].

The summary of the pros and cons of each kind of nanotechnology evaluated in GBM research is presented in Table 1 below.

The development of NanoTherm technology paved the way for other nanotherapy approvals for GBM treatment. Generally, there are several nanodrug therapies approved for the treatment of cancer and the list is growing [207]. For instance, liposomal DOX (registered under the names Doxil, Caelyx, Myocet, and Lipo-Dox) and nanoparticle-bound PTX (Abraxane^®^, developed by Abraxis/Celgene) are already approved for the treatment of metastatic breast cancer [112]. Liposomal DOX is also approved for ovarian cancer [112]. Pazenir^®^, produced by Ratiopharm GmbH, is PTX-formulated as albumin-bound nanoparticles. It received EMA approval in 2019 for the treatment of metastatic breast cancer, metastatic adenocarcinoma of the pancreas, and non-small cell lung cancer. Another approved drug is Vyxeos liposomal (Celator/Jazz Pharma), which contains daunorubicin and cytarabine and is indicated for the treatment of newly diagnosed therapy-related acute myeloid leukemia (t-AML) or AML with myelodysplasia-related changes (AML-MRC) in adults and pediatric patients 1 year and older. Onivyde (Merrimack Pharma) contains liposomal irinotecan and is registered for colorectal and pancreatic cancer treatment. Mepact^®^ (liposomal mifamurtide) is an immunomodulator with anti-tumor effects, registered for the treatment of osteosarcoma. Hensify (NBTXR3), developed by Nanobiotix, is a first-in-class radioenhancer. It is an aqueous suspension of crystalline hafnium oxide (HfO2) nanoparticles designed for injection directly into a tumor prior to radiotherapy [208]. Hensify^®^(NBTXR3) received European market approval (CE mark) enabling commercialization in 27 European Union countries for the treatment of locally advanced soft tissue sarcoma. Taking into consideration that currently there are several approved cancer drug therapies based on nanotechnology, we can assume that in the close future, new anti-GBM nanotherapies will be also developed, giving patients hope for successful therapy outcomes.

## Figures and Tables

**Table 1:** Nanoparticle characteristics by their pros and cons.

Type of NPs	Advantages	Disadvantages
Polymer-based nanoformulations	Enhanced ability to control the kinetics of drug release—gradually and over a longer period of time Can be modified to increase affinity to specific tissues or cells Created from biocompatible and biodegradable materials Easily modified surface to allow targeting, increase stability, and reduce immunogenicity	Large-scale production can be difficult and expensive Batch-to-batch variability may affect efficacy and safety Difficulty in penetrating deep tumor areas
Solid lipid nanoparticles	Composed mainly of natural materials, making them biocompatible, biodegradable, and safe for therapeutic use Their structure allows controlled drug release, reducing toxicity and increasing therapeutic efficacy High encapsulation efficiency for macromolecules such as siRNA and mRNA Surface functionalization with targeting ligands and surfactant coatings, improve uptake and access to the brain	Tendency to fuse (in the absence of PEG coating), leading to the leakage of encapsulated substances from lipid vesicles and increasing dispersibility Low, limited drug loading capacity Drug displacement caused by crystallization
Nanostructured lipid carriers	Higher drug loading capacity compared to SLN—greater versatility for a variety of therapeutic compounds Increased stability compared to SLN NLCs are less susceptible to drug shedding caused by crystallization during storage than SLNs Biocompatibility and biodegradability	Large-scale production can be difficult and expensive
Liposomes	Versatility in relation to encapsulation of hydrophilic as well as hydrophobic drugs Biocompatibility and biodegradability High potential for surface modification to improve biodistribution and pharmacokinetic properties Targeted delivery due to functionalization with ligands, e.g., CPP or antibody	Susceptible to oxidation and hydrolysis, which may lead to reduced therapeutic efficacy Rapid removal from the bloodstream by RES, which may limit the ability to reach the target site
Lipid nanocapsules	Functionalization of LNCs with peptides (e.g., NFL), enables targeted drug delivery to GBM cells, increasing the efficacy of therapy and minimizing negative effects on healthy tissues Internalization of LNCs into GBM cells Biocompatibility and biodegradability	Limited number of clinical studies evaluating their safety and efficacy in the treatment of GBM Production of LNCs on an industrial scale can be complex and costly Need for further in vivo studies to unequivocally confirm the efficacy and safety of LNC in the treatment of GBM
Nanodiscs	Like liposomes, they can increase the solubility and stability of drugs (especially important for hydrophobic drugs) Can improve the therapeutic index of drugs by increasing their concentration at the target site (tumor) and decreasing their concentration in healthy tissues Can be functionalized with ligands, allowing the targeting of specific cells Can be administered subcutaneously or directly into the tumor, which is less invasive than other delivery methods such as intravenous administration Nanodiscs, due to their HDL-mimicking properties, have a longer circulation time compared to other nanoparticles such as liposomes They combine the advantages of liposomes, such as biocompatibility and the ability to encapsulate drugs, with the advantages of nanoparticles, such as small size and the ability to functionalize with ligands	In some cases, nanodiscs need to be administered intracranially to ensure effective drug delivery to the brain, which is an invasive procedure Limited number of clinical trials evaluating their long-term safety and efficacy in the treatment of GBM
Dendrimers	Large capacity and functionality Targeted delivery possible due to high modifiability Anti-inflammatory properties—supportive in cancer therapy Possibility of controlled release of the drug	Can be rapidly removed from the bloodstream by the RES, which shortens their circulation time and limits their ability to reach their target site Difficult to produce
Gold nanoparticles	Ability to cross the BBB by passive diffusion through endothelial cells Can be functionalized with a variety of ligands, such as CPPs, allowing them to target delivery of drugs and other therapeutic compounds Potential for photothermal therapy (strong near-infrared absorption properties) Can also be used for imaging cancer cells Biocompatibility	Rapid removal from the bloodstream by the RES, which shortens their circulation time and limits their ability to reach their target site Producing nanoparticles with high purity and controlled properties can be expensive
Iron nanoparticles	Delivery of a wide range of drugs and other therapeutic compounds to cancer cells, including nucleic acids such as microRNAs Can be used for fluorescence imaging Safety and biocompatibility Magneticity, which allows for imaging and thermotherapy	May cause cytotoxicity in higher concentrations Difficult to control biodistribution
Silica nanoparticles	Large surface area and adjustable porosity Excellent biocompatibility Lower cost of production compared to other nanoparticles	Limited targetability May cause long-term toxicity related to their long half-life time
Exosomes	Low immunogenicity Biocompatibility Excellent, inherent targeting properties Ability to carry molecules with different charges	Acquisition can be difficult and expensive The development of scalable and efficient purification methods is essential for the wider use of exosomes in therapeutics Low loading efficiency Lack of long-term studies Batch-to-batch variability depending on source and isolation method
Macrophage cell membrane-based nanoparticles	High biocompatibility Decrease immunogenic clearance Adaptation to the tumor microenvironment	Batch-to-batch variability There is a potential risk that nanoparticles based on macrophage cell membranes may activate M2 macrophages in the tumor microenvironment, paradoxically promoting tumor growth High cost of production and hard to scale Limited knowledge of long-term effects
Virus-like particles	Biocompatibility High specificity VLPs are produced recombinantly, ensuring their uniformity in size and shape Cost-effective production Low toxicity	Limited knowledge of long-term effects

## Abbreviations

AMT	adsorption-mediated transcytosis
ApoE	apolipoprotein E
AuNPs	gold nanoparticles
BBB	blood–brain barrier
BBTB	blood–brain tumor barrier
BCECs	brain capillary endothelial cells
BEV	bevacizumab
BIOT-NFL	biotynylated neurofilament low subunit
BSA	bovine serum albumin
CED	convection-enhanced delivery
CMT	carrier-mediated transcytosis
CNS	central nervous system
CPPs	cell-penetrating peptides
DMF	dimethylformamide
DTX	docetaxel
DOX	doxorubicin
EGFR	epidermal growth factor receptor
EP-1	EGFR-targeting peptide
EphA	erythropoietin-producing human hepatocellular receptor A, ephrin type-A receptor
EPR	enhanced permeability and retention
ERLO	erlotinib
GBM	glioblastoma multiforme
GLUT1	glucose transporter 1
GSCs	glioblastoma stem cells
HER	human epidermal receptor
ICV	intracerebroventricular administration
InsR	insulin receptor
IONPs	iron oxide nanoparticles
LAT1	L-amino acids transporter 1
LDLR	low-density lipoprotein receptor
LNCs	lipid nanocapsules
LNPs	lipid nanoparticles
MGMT	O6-Methylguanine-DNA Methyltransferase
MTf	melanotransferrin
NDDSs	nanodrug delivery systems
NFL	neurofilament low subunit
NLCs	nanostructured lipid carriers
NPs	nanoparticles
PAMAM	polyamidoamine
PLGA	poly-lactic-co-glycolic acid
PNPs	polymer-based nanoparticles
PPV	poly[2-methoxy-5-(2-ethylhexyloxy)-p-phenylenevinylene]
PTX	paclitaxel
PVA	polyvinyl alcohol
RES	reticuloendothelial system
RMT	receptor-mediated transcytosis
SLNs	solid lipid nanoparticles
SPIONs	superparamagnetic iron oxide nanoparticles
TAMCs	tumor associated myeloid cells
TAMs	tumor-associated macrophages
Tf	transferrin
TJs	tight junctions
TMZ	temozolomide
TTFields	Tumor treating fields
VEGF	vascular endothelial growth factor
VIN	vincristine
VLPs	virus-like nanoparticles
